# From HPV genotypes to cervical lesion burden: public health-relevant heterogeneity across 1.29 million screening records in Guangdong, China

**DOI:** 10.3389/fpubh.2026.1897790

**Published:** 2026-09-17

**Authors:** Qian Zhao, Chenlu Yang, Qiaowen Bu, Xiaoli Sun, Guiying Hu, Lulu He, Zhifu Wei, Gongye Wang, Xiaowan Luo, Jiating Gu, Li Wu, Xiping Luo, Xiaoshan Hong

**Affiliations:** 1 Department of Gynecology, Guangdong Women and Children Hospital, Guangzhou, China; 2 Women and Children's Hospital, Southern University of Science and Technology, Guangzhou, China; 3 Department of Gynecology, The Affiliated Shunde Hospital of Jinan University, Foshan, China; 4 Graduate School, Guangzhou Medical University, Guangzhou, China; 5 Department of Gynecology, Boai Hospital of Zhongshan, Zhongshan, China; 6 Department of Gynecology, Jieyang People's Hospital, Jieyang, China

**Keywords:** cervical cancer, cervical lesions, HPV genotype, human papillomavirus, screening history

## Abstract

**Objective:**

To characterize the association between reported human papillomavirus (HPV) genotype detection patterns with cervical lesion burden and to assess variation by region, year, age, reported prior screening history, and diagnostic category in a province-wide cervical cancer screening program in Guangdong, China.

**Methods:**

We conducted a screening program-based cross-sectional study using 1,291,583 individual-level screening records collected during 2017–2022. Aggregate lesion data from Guangzhou, Dongguan, and Zhongshan supplemented city- and region-level lesion analyses. Genotype-specific analyses included 1,244,766 records with genotype-level results. Joinpoint regression and adjusted generalized linear models were used to assess temporal and age-related patterns. Sensitivity analyses retained only the earliest eligible record for each identifiable woman.

**Results:**

High-risk HPV was detected in 63,004 records (4.88%). Low-grade squamous intraepithelial lesions (LSIL), high-grade/microinvasive cervical lesions, and cervical cancer were recorded in 11,106 (0.86%), 6,633 (0.51%), and 382 (0.03%) records, respectively. The cities with the highest pooled proportions differed across lesion categories. Overall low-risk and high-risk HPV positivity showed significant positive annual percentage changes of 19.21 and 8.05%, respectively, whereas no individual high-risk genotype changed significantly. High-risk HPV positivity was highest in Northern Guangdong (5.6%), and HPV16 was the most frequently reported genotype in all four regions. Compared with participants aged <45 years, those aged ≥65 years had adjusted relative risks of 1.52 (95% CI, 1.22–1.91) for high-grade/microinvasive cervical lesions, 5.02 (2.43–10.35) for cervical cancer, and 1.51 (1.39–1.63) for high-risk HPV positivity. Age-related differences in severe lesions were more evident among records with no prior screening reported. The detection proportion of HPV16 increased from 28.6% in LSIL to 50.0% in high-grade/microinvasive cervical lesions and 69.3% in cervical cancer. HPV18 ranked second in cancer (18.3%), whereas HPV52 and HPV58 were more frequent in LSIL and high-grade/microinvasive lesions.

**Conclusion:**

This province-wide screening study linked reported HPV genotype patterns with cervical lesion burden and identified uneven disease burden across Guangdong, a higher burden of severe cervical lesions among older participants with no prior screening reported, and distinct genotype-specific detection patterns across lesion grades. These findings support locally adapted screening and follow-up strategies that prioritize high-burden regions, under-screened older women, and genotype-informed follow-up of HPV-positive women.

## Introduction

Cervical cancer remains a major public health challenge worldwide and continues to impose a substantial disease burden in China (1, 2). Persistent infection with high-risk human papillomavirus (HPV) is the principal cause of cervical precancerous lesions and cervical cancer (3). Although HPV vaccination and cervical cancer screening have strengthened cervical cancer prevention, HPV infection and cervical lesion burden remain unevenly distributed across regions and population groups. Local evidence derived from clearly defined screening settings is therefore needed to characterize screening-detected burden and support context-specific prevention strategies.

The prevalence and genotype distribution of HPV infection vary substantially by geographic region (4). Globally, HPV16 and HPV18 account for the largest proportion of invasive cervical cancers (5). However, studies from China and other Asian populations have shown that HPV52 and HPV58 are also highly prevalent and are frequently detected in precancerous lesions (6, 7). In addition, HPV genotype distribution may differ across lesion grades, age groups, and screening populations. For example, HPV16 is enriched in high-grade squamous intraepithelial lesions (HSIL) and cervical cancer, HPV prevalence in China shows a second peak among older women, and HPV positivity and genotype profiles may differ between gynecology clinic patients and women undergoing routine health examinations (6, 8–11). These differences highlight the need to interpret genotype patterns in relation to both lesion severity and screening context. Such evidence may inform the evaluation of vaccine-relevant genotype coverage, screening triage, and follow-up prioritization among HPV-positive women.

Guangdong Province provides an important setting for studying cervical cancer prevention in China. As one of the most populous provinces in the country, Guangdong accounts for a substantial proportion of China’s female population and has marked internal heterogeneity in economic development, population structure, urbanization, healthcare resources, and geographic environment. The Pearl River Delta is highly urbanized and economically developed, whereas eastern, western, and northern Guangdong include areas with different demographic and healthcare profiles. These differences may influence screening participation, HPV infection patterns, and cervical lesion detection. However, province-wide evidence from Guangdong remains limited, particularly studies that integrate HPV genotype distribution and cervical lesion burden with analyses of regional variation, temporal patterns, age-specific patterns, and prior screening history within the same screening framework.

Using data from a large-scale cervical cancer screening program in Guangdong Province, China, this study aimed to move beyond descriptive HPV prevalence by characterizing cervical lesion and HPV patterns across regions, calendar years, age groups, prior screening histories, and diagnostic categories, and by linking genotype-specific HPV detection with cervical lesion burden. We sought to identify public health-relevant heterogeneity within a province-wide screening framework and to generate evidence for locally adapted cervical cancer prevention in Guangdong and other socioeconomically diverse settings.

## Methods

### Study design and data source

We conducted a screening program-based cross-sectional study using routine data collected during 2017–2022 under the Guangdong “Two Cancers” screening initiative. The primary individual-level dataset comprised 1,291,583 screening records from 16 prefecture-level cities, 106 counties/districts, 514 townships/streets, and a total of 878 medical institutions. Screening was provided through maternal and child health hospitals, community health centers, or general hospitals.

Additional annual aggregate lesion counts and screening denominators from Guangzhou, Dongguan, and Zhongshan were used only to supplement city-level spatial and temporal analyses of cervical lesions. These aggregate data were not included in individual-level analyses of age, reported prior screening history, HPV positivity, cervical diagnostic category, or HPV genotype. All analyses were restricted to 2017–2022. City-year lesion data were unavailable for Dongguan in 2018 and 2019 and for Foshan and Jiangmen in 2017, and missing observations were not imputed.

### Study population, analysis unit, age groups, and regions

Screening records were eligible when age, region, cervical diagnostic status, and overall HPV status were available. The screening record was the primary unit of analysis. Because some women participated more than once during the study period, one woman could contribute more than one record to the primary analysis.

Age was categorized into four age groups: <45 years, 45–59 years, 60–64 years, and ≥65 years. The grouping reflected the screening program’s target age range and the study objective of evaluating outcomes among participants beyond the program’s standard upper age limit (12). The ≥65-year group was designated as the index comparison group for age-stratified analyses. Findings were interpreted as screening-detected proportions among participants rather than population prevalence.

Guangdong Province was divided into four geographic regions: eastern Guangdong, including Chaozhou, Shantou, Jieyang, and Shanwei; western Guangdong, including Zhanjiang, Maoming, Yangjiang, and Yunfu; northern Guangdong, including Shaoguan, Qingyuan, Meizhou, and Heyuan; and the Pearl River Delta, including Guangzhou, Foshan, Dongguan, Huizhou, Zhongshan, Jiangmen, and Zhaoqing.

### Screening and diagnostic procedures

According to the Guangdong provincial screening protocol, cervical cancer screening included gynecological examination, vaginal secretion examination, HPV testing, and ThinPrep cytology testing. Women who tested positive for HPV16 or HPV18, had suspicious or abnormal cytology results, or had abnormal findings on visual or gynecological examination were referred for colposcopy. Histopathological examination was performed when colposcopy showed abnormal or suspicious findings and was used as the diagnostic reference standard.

Cervical findings were classified according to the final diagnosis recorded in the provincial screening database. For analysis, the original diagnoses were recoded into mutually exclusive categories: normal cervix, inflammation, LSIL, high-grade/microinvasive cervical lesions, cervical cancer, and other diagnoses. Inflammation comprised inflammatory findings without evidence of LSIL, high-grade/microinvasive cervical lesions, or cervical cancer. High-grade/microinvasive cervical lesions comprised HSIL, adenocarcinoma *in situ* (AIS), and microinvasive squamous cell carcinoma or adenocarcinoma (MIC). This category was abbreviated as HSIL/AIS/MIC in tables and figures. Cervical cancer comprised invasive squamous cell carcinoma or adenocarcinoma not recorded as microinvasive disease. Other diagnoses included koilocytosis, cervical polyps, and condyloma. All participating institutions followed the provincial implementation protocol and relevant national technical guidelines for specimen collection, cytological and histopathological assessment, and data reporting.

### HPV testing and genotype framework

HPV testing was conducted using routine laboratory workflows at participating institutions. Because HPV assays and laboratory platforms were not uniform across centers, HPV results recorded in the provincial database were reviewed and harmonized using a common analytic framework to improve cross-center comparability. Overall low-risk and high-risk HPV positivity was analyzed in all 1,291,583 screening records. Genotype-specific analyses were restricted to 1,244,766 records with identifiable genotype-level results; the remaining 46,817 records reported only aggregate low-risk or high-risk HPV results and were therefore excluded from genotype-specific analyses. Available genotype-level results were harmonized into a common framework comprising 15 oncogenic or potentially oncogenic types (HPV16, 18, 31, 33, 35, 39, 45, 51, 52, 53, 56, 58, 59, 66, and 68) and six low-risk types (HPV6, 11, 42, 43, 44, and 81).

### Outcomes and prior screening history

The main outcomes were screening-detected proportions of cervical inflammation, LSIL, high-grade/microinvasive cervical lesions, cervical cancer, low-risk HPV positivity, and high-risk HPV positivity. Genotype-specific detection was summarized by diagnostic category and geographic region.

Prior screening history was obtained by staff interview at the current screening visit. Records were classified as no prior screening reported if participants reported no previous cervical cancer screening or related cervical examination, and as prior screening reported if any such examination had occurred before the current visit. This self-reported variable was distinct from repeated participation in the 2017–2022 program, which was identified using repeated participant identifiers and examined separately in the deduplication sensitivity analysis.

### Statistical analysis

Categorical variables were summarized as counts and percentages. Age-group differences were assessed using the chi-square test or Fisher’s exact test, as appropriate. For pairwise comparisons, the groups aged <45 years, 45–59 years, and 60–64 years were each compared with the prespecified group aged ≥65 years. After Bonferroni correction, a two-sided *p-*value <0.017 was considered statistically significant.

City-level pooled lesion proportions were calculated by dividing total lesion counts by total screening records during 2017–2022 and were expressed per 100,000 screening records. Annual provincial and regional lesion proportions were calculated using the corresponding annual counts and denominators. Joinpoint regression was used to estimate annual percentage changes (APCs) from crude, non-age-standardized annual proportions. Overall low-risk and high-risk HPV analyses used all screening records within each year, whereas genotype-specific analyses used records with genotype-level results. Because contributing cities varied across years, lesion trends and APC estimates were interpreted descriptively.

Continuous age-response relationships were estimated using generalized linear models with natural splines. Three degrees of freedom were selected according to the Akaike information criterion. The reference age for each spline model was determined from the corresponding analytic sample. Adjusted relative risks and 95% confidence intervals across age groups were estimated using separate generalized linear models, with participants aged <45 years as the reference group. Models were adjusted for contraceptive use, ethnicity, menopausal status, parity, reported prior screening history, urban–rural residence, and screening year.

In the sensitivity analysis, repeated participation was defined as more than one eligible record with the same participant identifier during 2017–2022. Only the earliest eligible record was retained for each identifiable woman, and records without participant identifiers were excluded. Age-specific descriptive comparisons, analyses stratified by reported prior screening history, and adjusted age-group models were then repeated.

Joinpoint analyses were performed using Joinpoint Regression Program version 4.8.0.1, and all other analyses were performed using R version 4.2.3. Unless otherwise specified, a two-sided *p-*value <0.05 was considered statistically significant.

## Results

### Study population and overall diagnostic profile

The primary analysis included 1,291,583 screening records collected during 2017–2022. Of these, 435,343 were from participants aged <45 years, 726,667 were from those aged 45–59 years, 103,799 were from those aged 60–64 years, and 25,774 from those aged ≥65 years.

Most records had normal cervical findings (1,263,800/1,291,583, 97.85%). Inflammation, LSIL, high-grade/microinvasive cervical lesions, and cervical cancer were recorded in 9,117 (0.71%), 11,106 (0.86%), 6,633 (0.51%), and 382 (0.03%) records, respectively. Low-risk and high-risk HPV positivity was recorded in 3,861 (0.30%) and 63,004 (4.88%) records, respectively (Table 1).

**Table 1:** Age-specific distribution of cervical diagnoses and HPV positivity across screening records in Guangdong Province, 2017–2022.

Characteristic		Age group							
		Overall	<45	P_1_	45–59	P_2_	60–64	P_3_	≥65
Screening records, *n*		1,291,583	435,343		726,667		103,799		25,774
Diagnosis (%)	Normal cervix	1,263,800 (97.85)	426,634 (98.00)	<0.001	711,025 (97.85)	<0.001	101,037 (97.34)	0.597	25,104 (97.40)
	Inflammation	9,117 (0.71)	2,617 (0.60)	<0.001	5,189 (0.71)	<0.001	1,062 (1.02)	0.433	249 (0.97)
	LSIL	11,106 (0.86)	3,802 (0.87)	0.124	6,199 (0.85)	0.221	904 (0.87)	0.166	201 (0.78)
	HSIL/AIS/MIC	6,633 (0.51)	2,112 (0.49)	<0.001	3,700 (0.51)	<0.001	645 (0.62)	0.285	176 (0.68)
	Cervical cancer	382 (0.03)	62 (0.01)	<0.001	241 (0.03)	<0.001	59 (0.06)	0.286	20 (0.08)
	Others	545 (0.04)	116 (0.03)	<0.001	313 (0.04)	<0.001	92 (0.09)	0.829	24 (0.09)
Low-risk HPV (%)	Negative	1,287,722 (99.70)	434,211 (99.74)		724,436 (99.69)		103,401 (99.62)		25,674 (99.61)
	Positive	3,861 (0.30)	1,132 (0.26)	<0.001	2,231 (0.31)	0.025	398 (0.38)	0.960	100 (0.39)
High-risk HPV (%)	Negative	1,228,579 (95.12)	415,307 (95.40)		691,680 (95.19)		97,395 (93.83)		24,197 (93.88)
	Positive	63,004 (4.88)	20,036 (4.60)	<0.001	34,987 (4.81)	<0.001	6,404 (6.17)	0.771	1,577 (6.12)

Values are *n* (%) unless otherwise indicated. Percentages were calculated using the corresponding column totals. Participants could contribute multiple screening records. P_1_, P_2_, and P_3_ compare the <45-, 45–59-, and 60–64-year groups, respectively, with the ≥65-year group. *p*-values were calculated using the chi-square test or Fisher’s exact test, as appropriate; after Bonferroni correction, *p* < 0.017 was considered statistically significant. Low- and high-risk HPV status followed the classification recorded in the screening database. AIS, adenocarcinoma in situ; HPV, human papillomavirus; HSIL, high-grade squamous intraepithelial lesion; LSIL, low-grade squamous intraepithelial lesion; MIC, microinvasive squamous cell carcinoma or adenocarcinoma.

### Lesion-category-specific geographic heterogeneity

Pooled city-level screening-detected proportions during 2017–2022 varied across Guangdong, and the leading cities differed by lesion category. For LSIL, the highest proportions were observed in Shaoguan (1,561 per 100,000 screened records), Yunfu (1,420), Maoming (1,378), and Huizhou (1,268). For high-grade/microinvasive cervical lesions, the highest proportions were observed in Maoming (857), Meizhou (669), Shaoguan (606), and Yunfu (605). Cervical cancer proportions were highest in Maoming (72), Heyuan (43), Shaoguan (37), and Jiangmen (36). Although Maoming and Shaoguan appeared among the leading cities in more than one category, the leading cities were not identical across LSIL, high-grade/microinvasive cervical lesions, and cervical cancer (Figure 1).

**Figure 1:**
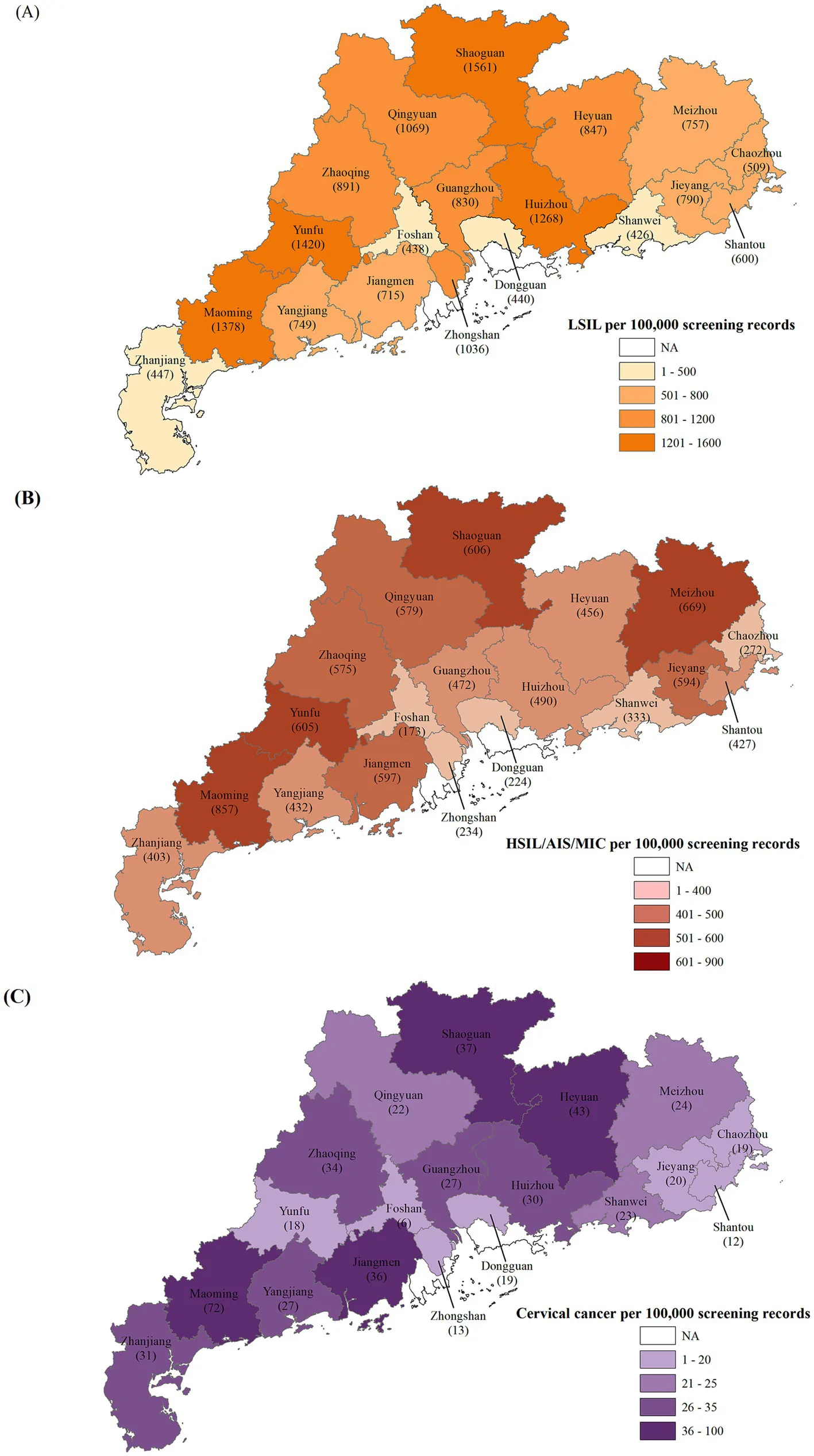
Pooled crude screening-detected proportions of cervical lesions across cities in Guangdong Province, 2017–2022. **(A)** LSIL, **(B)** HSIL/AIS/MIC, and **(C)** Cervical cancer. Values are expressed per 100,000 screening records and were calculated from available pooled lesion counts and screening denominators. Values for Guangzhou, Dongguan, and Zhongshan were derived from supplementary annual aggregate data; values for all other cities were derived from the individual-level primary dataset. Color scales are panel-specific. AIS, adenocarcinoma *in situ*; HSIL, high-grade squamous intraepithelial lesion; LSIL, low-grade squamous intraepithelial lesion; MIC, microinvasive squamous cell carcinoma or adenocarcinoma; NA, not available.

### Temporal patterns in cervical lesions and HPV positivity

The expanded city-year lesion dataset comprised 2,289,337 screening records during 2017–2022, with annual denominators ranging from 230,399 to 626,613 records. At the provincial level, the crude LSIL proportion generally increased from 489.59 per 100,000 screening records in 2017 to 990.97 in 2022. The proportion of high-grade/microinvasive cervical lesions increased from 217.45 in 2017 to 404.08 in 2020 and then declined to 339.78 in 2022. The cervical cancer proportion decreased overall from 34.72 in 2017 to 21.83 in 2022, although annual values fluctuated (Supplementary Table S1, Supplementary Figure S3).

Regional patterns differed by lesion category. The observed LSIL proportions were higher in 2022 than in 2017 in all four regions, with the largest absolute increase in Northern Guangdong. In the Pearl River Delta, the observed proportion of high-grade/microinvasive cervical lesions increased from 131.46 per 100,000 screening records in 2017 to 270.82 in 2021 before declining to 169.48 in 2022. Cervical cancer proportions decreased overall in the Pearl River Delta, from 38.89 per 100,000 screening records in 2017 to 13.35 in 2022, whereas Western Guangdong had the highest proportions among the four regions in 2021 and 2022. (Figure 2, Supplementary Table S1). City-level trajectories varied substantially in both direction and magnitude, with no uniform temporal pattern across cities for either LSIL or high-grade/microinvasive cervical lesions (Supplementary Figures S1, S2). The underlying city-year values are provided in Supplementary Table S2. Provincial fitted trends were broadly consistent with the observed annual patterns described above (Supplementary Figure S3). Because the set of contributing cities was not completely constant across years, all lesion-related temporal patterns were interpreted descriptively.

**Figure 2:**
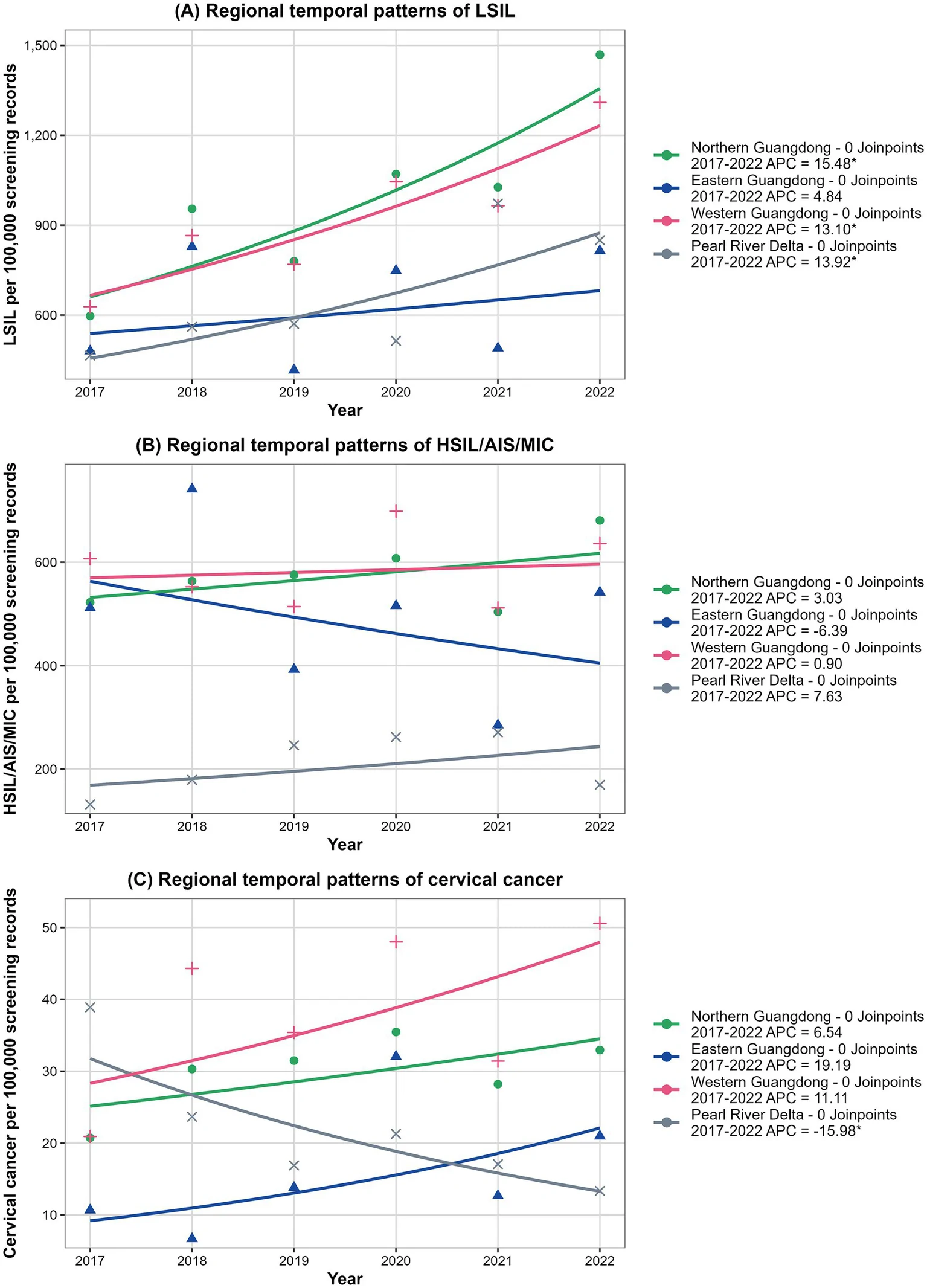
Annual crude screening-detected proportions of cervical lesions across regions of Guangdong Province, 2017–2022. **(A)** LSIL, **(B)** HSIL/AIS/MIC, and **(C)** cervical cancer. Points represent observed annual proportions, and lines represent fitted Joinpoint trends. APC estimates are shown in the panel legends; asterisks indicate *p* < 0.05. Values are expressed per 100,000 screening records. APC, annual percentage change; AIS, adenocarcinoma *in situ*; HSIL, high-grade squamous intraepithelial lesion; LSIL, low-grade squamous intraepithelial lesion; MIC, microinvasive squamous cell carcinoma or adenocarcinoma.

In the primary individual-level dataset, overall low-risk and high-risk HPV positivity showed significant positive fitted APCs of 19.21% (95% CI, 4.18–44.34) and 8.05% (95% CI, 2.53–14.73), respectively. At the genotype-specific level, significant positive APCs were observed only for the low-risk types HPV6, HPV42, and HPV44; none of the individual high-risk HPV genotypes showed an APC significantly different from zero. Annual denominators for overall HPV positivity and genotype-specific analyses are reported separately in Supplementary Table S3.

### Age-related patterns of cervical lesions and HPV positivity

High-risk HPV positivity was 4.60, 4.81, 6.17, and 6.12% among participants aged <45 years, 45–59 years, 60–64 years, and ≥65 years, respectively. Compared with participants aged ≥65 years, positivity was significantly lower among those aged <45 years and 45–59 years but did not differ significantly among those aged 60–64 years. Low-risk HPV positivity showed a similar pattern (0.26, 0.31, 0.38, and 0.39%). After Bonferroni correction, only participants aged <45 years had significantly lower positivity than those aged ≥65 years. The proportions of high-grade/microinvasive cervical lesions were 0.49, 0.51, 0.62, and 0.68% across the four age groups, respectively. The corresponding cervical cancer proportions were 0.01, 0.03, 0.06, and 0.08%. Both outcomes were significantly less frequent among participants aged <45 years and 45–59 years than among those aged ≥65 years, whereas no significant difference was observed among participants aged 60–64 years (Table 1). Across regions, cervical cancer proportions generally increased with age. High-risk HPV positivity was also higher in the older age groups in most regions, although the magnitude and statistical significance of age-related differences varied geographically (Supplementary Table S4).

Adjusted analyses showed higher relative risks of high-grade/microinvasive cervical lesions, cervical cancer, and HPV positivity among participants aged ≥65 years. Compared with those aged <45 years, participants aged ≥65 years had adjusted relative risks of 1.52 (95% CI, 1.22–1.91) for high-grade/microinvasive cervical lesions, 5.02 (95% CI, 2.43–10.35) for cervical cancer, 2.42 (95% CI, 1.80–3.26) for low-risk HPV positivity, and 1.51 (95% CI, 1.39–1.63) for high-risk HPV positivity. For LSIL, the adjusted relative risks were 0.98 (95% CI, 0.94–1.03), 1.04 (95% CI, 0.96–1.13), and 1.00 (95% CI, 0.81–1.24) among participants aged 45–59 years, 60–64 years, and ≥65 years, respectively, compared with those aged <45 years. Adjusted continuous age-response curves and age-group estimates are presented together with the corresponding estimates from the primary and deduplicated analyses (Figure 3, Supplementary Table S5).

**Figure 3:**
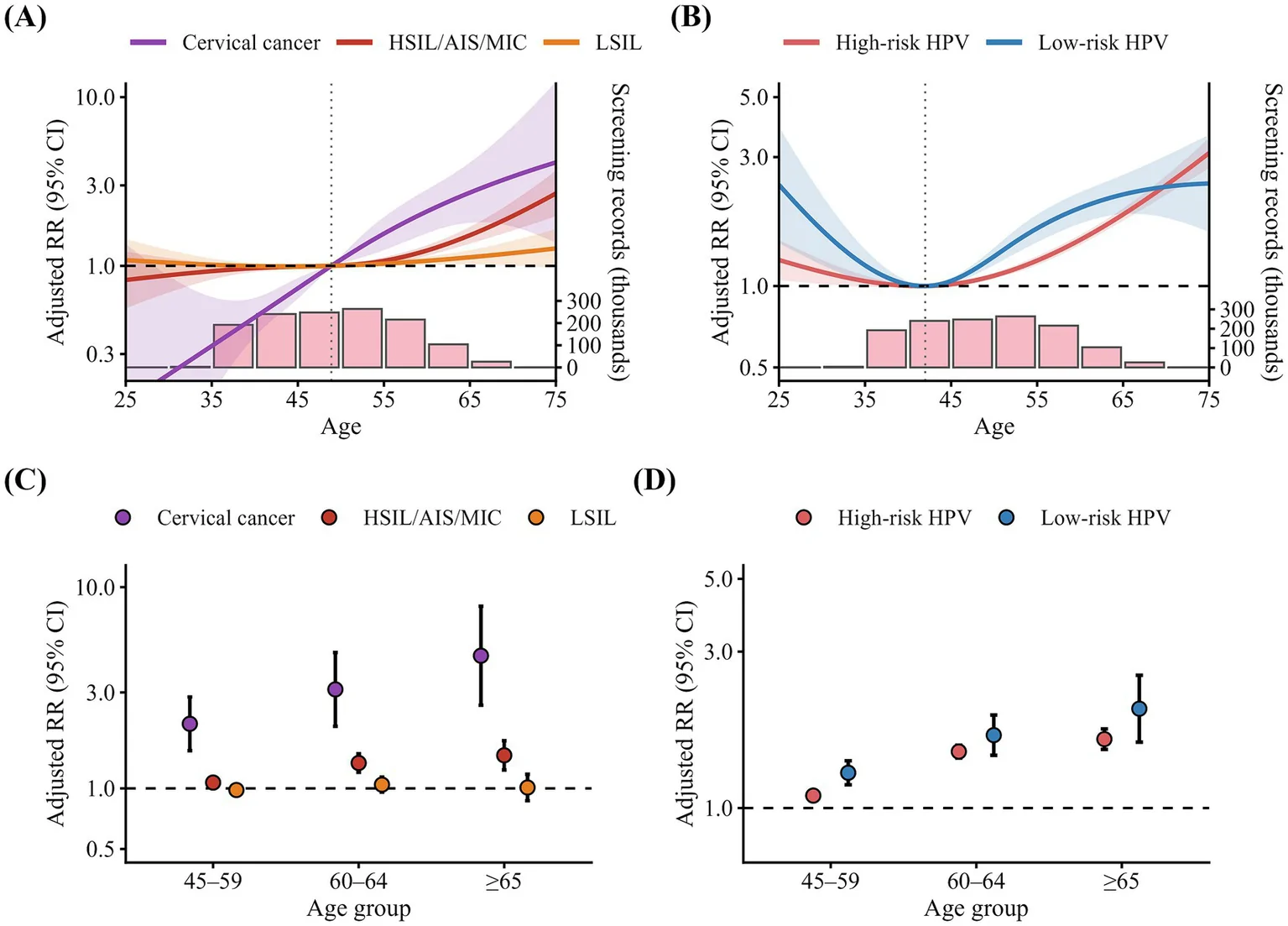
Adjusted associations of age with cervical lesions and HPV positivity among screening records in Guangdong Province, 2017–2022. **(A,B)** Adjusted age-response curves for cervical lesions and HPV positivity. **(C,D)** Adjusted age-group RRs. Lines and points represent RRs; Shaded areas and error bars indicate 95% CIs. Histograms show the age distribution of screening records. Reference RR was 1.0 at ages 48.9 years in panel A and age 43.0 years in panel B, and for participants aged <45 years in panels **(C)** and **(D)**. Models were adjusted for contraceptive use, ethnicity, menopausal status, parity, reported prior screening history, urban–rural residence, and screening year. AIS, adenocarcinoma *in situ*; CI, confidence interval; HPV, human papillomavirus; HSIL, high-grade squamous intraepithelial lesion; LSIL, low-grade squamous intraepithelial lesion; MIC, microinvasive squamous cell carcinoma or adenocarcinoma; RR, relative risk.

### Prior screening history and age-specific patterns

Among records with no prior screening reported, high-grade/microinvasive cervical lesions were recorded in 0.54, 0.58, 0.63, and 0.72% among the <45, 45–59, 60–64, and ≥65 years groups, respectively. The corresponding proportions of cervical cancer were 0.02, 0.04, 0.07, and 0.08%. Compared with participants aged ≥65 years, both outcomes were significantly less frequent among those aged <45 years and 45–59 years, whereas no significant difference was observed among those aged 60–64 years. High-risk HPV positivity was 4.61, 4.85, 6.12, and 6.14%, respectively, with the same pattern of pairwise differences.

Among records with prior screening reported, age-related differences in cervical lesion proportions were less evident. High-grade/microinvasive cervical lesions were recorded in 0.37, 0.34, 0.60, and 0.49% across the four age groups, and cervical cancer in 0.01, 0.02, 0.02, and 0.05%, respectively. None of these pairwise differences reached the Bonferroni-adjusted significance threshold. High-risk HPV positivity remained higher in the two older groups, with proportions of 4.59, 4.73, 6.36, and 6.02%, respectively (Table 2).

**Table 2:** Cervical diagnoses and HPV positivity by reported prior screening history and age group among screening records in Guangdong Province, 2017–2022.

Category		Age group						
		<45	P_1_	45–59	P_2_	60–64	P_3_	≥65
No prior screening reported, *n*		299,318		506,288		82,456		21,653
Diagnosis (%)	Normal cervix	293,169 (97.95)	<0.001	495,034 (97.78)	<0.001	80,273 (97.35)	0.999	21,080 (97.35)
	Inflammation	1,752 (0.59)	<0.001	3,564 (0.70)	<0.001	842 (1.02)	0.516	210 (0.97)
	LSIL	2,662 (0.89)	0.123	4,303 (0.85)	0.325	704 (0.85)	0.336	170 (0.79)
	HSIL/AIS/MIC	1,613 (0.54)	<0.001	2,957 (0.58)	0.013	516 (0.63)	0.127	156 (0.72)
	Cervical cancer	45 (0.02)	<0.001	200 (0.04)	0.005	54 (0.07)	0.384	18 (0.08)
	Others	77 (0.03)	<0.001	230 (0.05)	0.010	67 (0.08)	0.790	19 (0.09)
Low-risk HPV (%)	Negative	298,556 (99.75)	<0.001	504,734 (99.69)	0.018	82,135 (99.61)	0.807	21,566 (99.60)
	Positive	762 (0.25)		1,554 (0.31)		321 (0.39)		87 (0.40)
High-risk HPV (%)	Negative	285,529 (95.39)	<0.001	481,733 (95.15)	<0.001	77,409 (93.88)	0.924	20,324 (93.86)
	Positive	13,789 (4.61)		24,555 (4.85)		5,047 (6.12)		1,329 (6.14)
								
Prior screening reported, *n*		136,025		220,379		21,343		4,121
Diagnosis (%)	Normal cervix	133,465 (98.12)	0.032	215,991 (98.01)	0.103	20,764 (97.29)	0.204	4,024 (97.65)
	Inflammation	865 (0.64)	0.018	1,625 (0.74)	0.119	220 (1.03)	0.672	39 (0.95)
	LSIL	1,140 (0.84)	0.603	1,896 (0.86)	0.55	200 (0.94)	0.282	31 (0.75)
	HSIL/AIS/MIC	499 (0.37)	0.239	743 (0.34)	0.104	129 (0.60)	0.434	20 (0.49)
	Cervical cancer	17 (0.01)	0.106	41 (0.02)	0.187	5 (0.02)	0.317	2 (0.05)
	Others	39 (0.03)	0.009	83 (0.04)	0.023	25 (0.12)	0.999	5 (0.12)
Low-risk HPV (%)	Negative	135,655 (99.73)	0.544	219,702 (99.69)	0.887	21,266 (99.64)	0.774	4,108 (99.68)
	Positive	370 (0.27)		677 (0.31)		77 (0.36)		13 (0.32)
High-risk HPV (%)	Negative	129,778 (95.41)	<0.001	209,947 (95.27)	<0.001	19,986 (93.64)	0.421	3,873 (93.98)
	Positive	6,247 (4.59)		10,432 (4.73)		1,357 (6.36)		248 (6.02)

Values are *n* (%) unless otherwise indicated. Percentages were calculated using age-group totals within each reported prior screening-history stratum as denominators. Participants could contribute multiple screening records. Prior screening history was self-reported and distinct from repeated program participation. Within each stratum, P_1_, P_2_, and P_3_ compare the <45-, 45–59-, and 60–64-year groups, respectively, with the ≥65-year group. *p*-values were calculated using the chi-square test or Fisher’s exact test, as appropriate; after Bonferroni correction, *p* < 0.017 was considered statistically significant. Low- and high-risk HPV status followed the classification recorded in the screening database. AIS, adenocarcinoma in situ; HPV, human papillomavirus; HSIL, high-grade squamous intraepithelial lesion; LSIL, low-grade squamous intraepithelial lesion; MIC, microinvasive squamous cell carcinoma or adenocarcinoma.

### Genotype-specific HPV detection across cervical diagnostic categories

Genotype-level information was available for 1,244,766 screening records. Within this subset, reported high-risk HPV positivity was 2.1% among records with normal cervical findings, 73.1% with inflammation, 71.7% with LSIL, 79.5% with high-grade/microinvasive cervical lesions, and 91.8% with cervical cancer. HPV16 was the most frequently reported genotype overall, followed by HPV52, HPV18, and HPV58. HPV16 was detected in 28.6% of LSIL records, 50.0% of high-grade/microinvasive cervical lesion records, and 69.3% of cervical cancer records. HPV18 was the second most frequently reported genotype in cervical cancer, with a detection proportion of 18.3%. HPV52 and HPV58 were more frequently reported in LSIL (7.7 and 4.1%, respectively) and high-grade/microinvasive lesions (5.9 and 5.1%) than in cervical cancer (1.6% for each genotype) (Table 3).

**Table 3:** Reported HPV genotype detection by cervical diagnostic category among 1,244,766 screening records with genotype-level information.

HPV type	Total	Normal cervix	Inflammation	LSIL	HSIL/AIS/MIC	Cervical cancer	Others
Screening records, n	1,244,766	1,226,541 (98.54)	6,347 (0.51)	6,841 (0.55)	4,342 (0.35)	306 (0.02)	389 (0.03)
Low-risk HPV frame							
Positive	3,521 (0.3)	2,964 (0.2)	225 (3.5)	247 (3.6)	70 (1.6)	1 (0.3)	14 (3.6)
HPV6 (%)	925 (0.1)	744 (0.1)	80 (1.3)	64 (0.9)	30 (0.7)	0 (0.0)	7 (1.8)
HPV11 (%)	288 (0.0)	247 (0.0)	13 (0.2)	13 (0.2)	10 (0.2)	0 (0.0)	5 (1.3)
HPV42 (%)	607 (0.0)	495 (0.0)	50 (0.8)	50 (0.7)	10 (0.2)	0 (0.0)	2 (0.5)
HPV43 (%)	442 (0.0)	367 (0.0)	28 (0.4)	39 (0.6)	7 (0.2)	0 (0.0)	1 (0.3)
HPV44 (%)	480 (0.0)	411 (0.0)	23 (0.4)	36 (0.5)	8 (0.2)	1 (0.3)	1 (0.3)
HPV81 (%)	1,101 (0.1)	940 (0.1)	57 (0.9)	87 (1.3)	12 (0.3)	0 (0.0)	5 (1.3)
High-risk HPV frame							
Positive	39,770 (3.2)	26,226 (2.1)	4,637 (73.1)	4,908 (71.7)	3,450 (79.5)	281 (91.8)	268 (68.9)
HPV16 (%)	11,345 (0.9)	4,878 (0.4)	1,979 (31.2)	1,959 (28.6)	2,171 (50.0)	212 (69.3)	146 (37.5)
HPV18 (%)	5,665 (0.5)	2,501 (0.2)	1,280 (20.2)	1,254 (18.3)	502 (11.6)	56 (18.3)	72 (18.5)
HPV31 (%)	1,080 (0.1)	841 (0.1)	77 (1.2)	85 (1.2)	66 (1.5)	1 (0.3)	10 (2.6)
HPV33 (%)	1,239 (0.1)	968 (0.1)	77 (1.2)	92 (1.3)	93 (2.1)	0 (0.0)	9 (2.3)
HPV35 (%)	988 (0.1)	773 (0.1)	73 (1.2)	83 (1.2)	46 (1.1)	1 (0.3)	12 (3.1)
HPV39 (%)	1,785 (0.1)	1,481 (0.1)	126 (2.0)	135 (2.0)	40 (0.9)	0 (0.0)	3 (0.8)
HPV45 (%)	722 (0.1)	567 (0.0)	52 (0.8)	59 (0.9)	35 (0.8)	2 (0.7)	7 (1.8)
HPV51 (%)	2,116 (0.2)	1,662 (0.1)	181 (2.9)	195 (2.9)	68 (1.6)	1 (0.3)	9 (2.3)
HPV52 (%)	6,586 (0.5)	5,330 (0.4)	443 (7.0)	528 (7.7)	256 (5.9)	5 (1.6)	24 (6.2)
HPV53 (%)	1,862 (0.1)	1,495 (0.1)	170 (2.7)	152 (2.2)	37 (0.9)	0 (0.0)	8 (2.1)
HPV56 (%)	1,707 (0.1)	1,373 (0.1)	125 (2.0)	151 (2.2)	48 (1.1)	1 (0.3)	9 (2.3)
HPV58 (%)	3,298 (0.3)	2,552 (0.2)	223 (3.5)	279 (4.1)	220 (5.1)	5 (1.6)	19 (4.9)
HPV59 (%)	1,360 (0.1)	1,128 (0.1)	89 (1.4)	94 (1.4)	45 (1.0)	1 (0.3)	3 (0.8)
HPV66 (%)	1,223 (0.1)	1,013 (0.1)	81 (1.3)	91 (1.3)	32 (0.7)	0 (0.0)	6 (1.5)
HPV68 (%)	2,454 (0.2)	2,037 (0.2)	175 (2.8)	164 (2.4)	66 (1.5)	2 (0.7)	10 (2.6)

Values are *n* (column %) unless otherwise indicated. The “Screening records, *n*” row shows the distribution of diagnostic categories within the genotype-level subset. Genotype-row percentages were calculated using the corresponding diagnostic-category totals. Records with multiple infections contributed once to each detected genotype. The analysis excluded 46,817 records without genotype-level results. AIS, adenocarcinoma in situ; HPV, human papillomavirus; HSIL, high-grade squamous intraepithelial lesion; LSIL, low-grade squamous intraepithelial lesion; MIC, microinvasive squamous cell carcinoma or adenocarcinoma.

### Regional variation in HPV genotype patterns

High-risk HPV positivity varied across the four regions, with proportions of 4.2% in Eastern Guangdong, 5.6% in Northern Guangdong, 4.9% in the Pearl River Delta, and 4.6% in Western Guangdong. Genotype-level information was available for 222,205, 354,105, 327,824, and 340,632 records in these regions, respectively. Among records with genotype-level information, HPV16 was the most frequently reported genotype in every region, with detection proportions ranging from 0.7 to 1.1%. HPV52, HPV18, and HPV58 were also consistently among the predominant genotypes, although their relative frequencies varied across regions (Supplementary Table S6).

### Sensitivity analyses

After retaining only the earliest eligible screening record for each identifiable woman, 24,120 subsequent records and 32 records without participant identifiers were excluded, leaving 1,267,431 records. Age-specific distributions and patterns by reported prior screening history remained consistent with the primary results (Supplementary Tables S7, S8).

## Discussion

In this province-wide study based on 1,291,583 cervical cancer screening records from Guangdong Province, we linked HPV genotype patterns with cervical lesion burden and identified public health-relevant heterogeneity across four main dimensions: lesion-grade-specific geographic heterogeneity, regional variation in HPV genotype patterns, age-related differences according to reported prior screening history, and genotype-specific HPV detection across cervical diagnostic categories. These findings suggest that cervical cancer prevention in Guangdong should not rely solely on overall HPV prevalence or province-level averages. Instead, regional burden, prior screening history, and genotype-specific lesion profiles should be considered when developing locally adapted screening and follow-up strategies.

The lesion-grade-specific geographic heterogeneity observed in this study is consistent with reported regional disparities in cervical cancer burden in China and internationally (13–15). In China, rural and western areas generally carry a higher burden than urban and eastern areas (13, 14), while international data also show greater cervical cancer burden in socioeconomically disadvantaged settings (15). Similar income-related disparities in cervical cancer incidence and stage at diagnosis have been reported in the United States (28). Within Guangdong, this broader pattern was partly reflected in the Pearl River Delta, which had the lowest pooled proportion of high-grade/microinvasive cervical lesions and generally lower lesion proportions than Northern and Western Guangdong. Importantly, the leading cities differed across LSIL, high-grade/microinvasive cervical lesions, and cervical cancer. This lesion-category-specific heterogeneity suggests that province-level averages may mask distinct local needs for screening, colposcopy, pathology, and follow-up.

Temporal patterns should be interpreted in the context of changes in screening participation and city-year data availability. In the expanded lesion dataset, screening records declined from 626,613 in 2020 to 467,199 in 2021 and 274,883 in 2022, although the contributing cities varied across years (Supplementary Table S1). Similar reductions in HPV screening and testing activity were reported during the COVID-19 pandemic. In Weifang, the number of HPV samples received in 2020, 2021, and 2022 was 24.03, 14.79, and 24.76% lower, respectively, than in 2019 (16). HPV screening visits also fell by approximately 50% during the outbreak in Wuhan (17). A global meta-analysis also reported a 51.8% reduction in cervical screening during the pandemic (18). Guangdong provincial monitoring subsequently documented a 12.68-percentage-point increase in cervical screening coverage from 2022 to 2023, providing local evidence of increased screening participation after the study period (19). Taken together, these findings support the possibility that pandemic-related changes in screening participation contributed to the temporal patterns observed in our study.

The regional genotype analysis identified geographic heterogeneity, although formal spatial clustering was not assessed. High-risk HPV positivity was highest in Northern Guangdong, whereas HPV16 was the most frequently reported genotype in all four regions. HPV52, HPV18, and HPV58 were also consistently among the predominant genotypes, although their relative frequencies varied geographically. This pattern was broadly consistent with a nationwide Chinese analysis and studies from northeastern Guangdong and the Pearl River Delta, which reported a shared group of predominant genotypes but different regional rankings (6, 20, 21). These findings suggest that regional variation in Guangdong mainly reflects differences in the relative frequencies of commonly detected genotypes. They provide local evidence for evaluating the regional genotype coverage of HPV vaccines. However, formal clustering and vaccine-effectiveness analyses would be required to support region-specific vaccine strategies.

The diagnostic-category-specific analysis further showed that overall genotype rankings did not fully reflect lesion-related importance. HPV16 detection increased progressively across LSIL, high-grade/microinvasive cervical lesions, and cervical cancer. This pattern was consistent with findings from Guizhou, where HPV16 increased from 21.7% in LSIL to 56.7% in cervical cancer (22). A Chongqing cohort also identified HPV16, HPV18, HPV52, and HPV58 as predominant in high-grade lesions and cervical cancer (23), while a histology-based study reported different genotype profiles between LSIL and HSIL (24). In our study, HPV18 ranked second in cervical cancer despite being less frequently reported overall than HPV52, whereas HPV52 and HPV58 were more prominent in precursor lesions. Thus, overall genotype frequency and cancer-related enrichment are not interchangeable measures.

Our age-stratified findings are consistent with evidence that cervical precancerous lesions may remain clinically relevant in some older women, particularly those with inadequate prior screening. A meta-analysis reported no clear decline in high-risk HPV prevalence among women over 50 years (25). In Denmark, a catch-up HPV testing study among women aged 65–69 years without previous HPV-based screening found a higher detection rate of cervical intraepithelial neoplasia grade 2 or worse (CIN2+) than standard care, supporting the potential value of targeted testing in insufficiently screened older women (26). Korean hospital-based data also reported higher detection of HSIL and ≥CIN3 among women aged ≥65 years than in younger groups (27). In our adjusted analyses, participants aged ≥65 years had higher relative risks of high-grade/microinvasive cervical lesions, cervical cancer, and HPV positivity than those aged <45 years, whereas no clear age-related association was observed for LSIL. In the stratified descriptive analyses, age-related differences in high-grade/microinvasive cervical lesions and cervical cancer were more evident among records with no prior screening reported than among those with prior screening reported. Together, these findings highlight prior screening history as an important consideration in screening decisions for older women and support prioritizing those with inadequate or unknown screening histories for catch-up or continued cervical screening beyond the routine upper age limit.

This study has several strengths, including its large sample size, province-wide coverage, and integration of regional, temporal, age-specific, screening-history-specific, and genotype-specific analyses within the same screening framework. This integrated approach allowed us to link HPV genotype patterns with cervical lesion burden and to identify heterogeneity with public health relevance. The robustness of the main age-related and prior-screening-history findings was further supported by sensitivity analyses retaining only the earliest eligible screening record for each identifiable woman. Guangdong is also an informative setting because of its large population and substantial internal socioeconomic diversity. Several limitations should also be acknowledged. First, the cross-sectional design precluded causal inference and assessment of cervical lesion progression. Second, the study included screening participants rather than a random provincial sample. The screening-detected proportions may have been overestimated if higher-risk women were more likely to participate, or underestimated if underserved high-risk women were underrepresented. Third, the use of aggregate data and changes in contributing cities could have shifted geographic and temporal estimates in either direction. Fourth, variation in HPV testing and genotype reporting across institutions may have exaggerated or attenuated regional and genotype-specific differences, while misclassification of self-reported prior screening history may have weakened contrasts between groups. Finally, genotype-specific findings reflected co-detection in records with multiple infections and did not establish type-specific causal attribution.

## Conclusion

This large-scale study linked reported HPV genotype detection patterns with cervical lesion burden across 1.29 million screening records in Guangdong Province. We identified public health-relevant heterogeneity across regions, age groups, reported prior screening histories, and cervical diagnostic categories. HPV16 showed progressively higher detection proportions across increasingly severe diagnostic categories, whereas HPV52 and HPV58 were more frequently detected in precursor lesions than in cervical cancer. Older participants with no prior screening reported had higher screening-detected proportions of high-grade/microinvasive cervical lesions and cervical cancer. These findings support locally adapted cervical cancer prevention strategies that prioritize high-burden regions, under-screened older women, and genotype-informed follow-up of HPV-positive women.

## Data Availability

The de-identified datasets supporting the conclusions of this article are available from the corresponding authors upon reasonable request.
