# Supplementary material for: From HPV genotypes to cervical lesion burden: public health-relevant heterogeneity across 1.29 million screening records in Guangdong, China
Source: Front Public Health. 2026 Sep 17;14:1897790. doi: 10.3389/fpubh.2026.1897790 (PMC13627325; doi:10.3389/fpubh.2026.1897790)
Supplement: Supplementary file 1 [file Data_Sheet_1.pdf]

Supplementary Tables

Table S1. Annual screening denominators, cervical lesion and HPV-positive counts, and crude screening-detected proportions by region, 2017-2022.

Region	Year	Screening records, n	LSIL, n	HSIL/AIS/MIC, n	Cervical cancer, n	HPV-tested records, n	Low-risk HPV positive, n	High-risk HPV positive, n	LSIL per100k	HSIL/AIS/MIC per100k	Cervical cancer per100k	Low-risk HPV positive per100k	High-risk HPV positive per100k
Total	2017	230399	1128	501	80	52410	132	2128	489.59	217.45	34.72	251.86	4060.29
Total	2018	242758	1648	789	65	108987	186	5215	678.87	325.02	26.78	170.66	4784.97
Total	2019	447485	2896	1784	109	335012	809	14307	647.17	398.67	24.36	241.48	4270.59
Total	2020	626613	4392	2532	178	339271	981	17408	700.91	404.08	28.41	289.15	5131
Total	2021	467199	4284	1620	95	328093	1343	14872	916.95	346.75	20.33	409.34	4532.86
Total	2022	274883	2724	934	60	127810	410	9074	990.97	339.78	21.83	320.79	7099.6
Eastern Guangdong	2017	9378	45	48	1	9378	39	420	479.85	511.84	10.66	415.87	4478.57
Eastern Guangdong	2018	14967	124	111	1	14967	11	934	828.49	741.63	6.68	73.5	6240.4
Eastern Guangdong	2019	50687	211	199	7	50687	170	2093	416.28	392.61	13.81	335.39	4129.26
Eastern Guangdong	2020	62408	467	322	20	62408	132	2136	748.3	515.96	32.05	211.51	3422.64
Eastern Guangdong	2021	63118	309	180	8	63118	224	2080	489.56	285.18	12.67	354.89	3295.41
Eastern Guangdong	2022	28608	233	155	6	28608	46	1850	814.46	541.81	20.97	160.79	6466.72
Northern Guangdong	2017	24114	144	126	5	24114	81	922	597.16	522.52	20.73	335.9	3823.51
Northern Guangdong	2018	32999	315	186	10	32999	138	1636	954.57	563.65	30.3	418.19	4957.73
Northern Guangdong	2019	85784	669	494	27	85784	442	4976	779.87	575.86	31.47	515.25	5800.62
Northern Guangdong	2020	98731	1057	600	35	98731	361	4975	1070.59	607.71	35.45	365.64	5038.94
Northern Guangdong	2021	92223	947	465	26	92223	590	5004	1026.86	504.21	28.19	639.75	5425.98
Northern Guangdong	2022	36419	535	248	12	36419	190	3045	1469.01	680.96	32.95	521.71	8361.02
Pearl River Delta	2017	182569	849	240	71	4580	1	162	465.03	131.46	38.89	21.83	3537.12
Pearl River Delta	2018	156423	877	280	37	22652	3	862	560.66	179	23.65	13.24	3805.4

Pearl River Delta	2019	189493	1081	466	32	77020	74	3055	570.47	245.92	16.89	96.08	3966.5
Pearl River Delta	2020	375891	1932	984	80	88549	364	4841	513.98	261.78	21.28	411.07	5467.03
Pearl River Delta	2021	257735	2506	698	44	118629	475	5891	972.32	270.82	17.07	400.41	4965.9
Pearl River Delta	2022	172288	1464	292	23	25215	84	1758	849.74	169.48	13.35	333.14	6972.04
Western Guangdong	2017	14338	90	87	3	14338	11	624	627.7	606.78	20.92	76.72	4352.07
Western Guangdong	2018	38369	332	212	17	38369	34	1783	865.28	552.53	44.31	88.61	4646.98
Western Guangdong	2019	121521	935	625	43	121521	123	4183	769.41	514.31	35.38	101.22	3442.2
Western Guangdong	2020	89583	936	626	43	89583	124	5456	1044.84	698.79	48	138.42	6090.44
Western Guangdong	2021	54123	522	277	17	54123	54	1897	964.47	511.8	31.41	99.77	3504.98
Western Guangdong	2022	37568	492	239	19	37568	90	2421	1309.63	636.18	50.57	239.57	6444.31

Cervical lesion proportions were calculated using the corresponding annual number of screening records as the denominator, whereas HPV positivity proportions were calculated using the annual number of HPV-tested records. All proportions were crude, expressed per 100,000 records, and were not age standardized. The dataset combined individual-level program data with available aggregate city-year lesion data from Guangzhou, Dongguan, and Zhongshan. Contributing cities varied across years, and missing city-year observations were not imputed. AIS, adenocarcinoma in situ; HPV, human papillomavirus; HSIL, high-grade squamous intraepithelial lesion; LSIL, low-grade squamous intraepithelial lesion; MIC, microinvasive squamous cell carcinoma or adenocarcinoma.

Table S2. City-year screening denominators, cervical lesion and HPV-positive counts, and crude screening-detected proportions, 2017-2022.

City	Year	Screening records, n	LSIL, n	HSIL/AIS/MIC, n	Cervical cancer, n	HPV-tested records, n	Low-risk HPV positive, n	High-risk HPV positive, n	LSIL per100k	HSIL/AIS/MIC per100k	Cervical cancer per100k	Low-risk HPV positive per100k	High-risk HPV positive per100k
Chaozhou	2017	337	2	1	0	337	0	43	593.47	296.74	0	0	12759.64
Chaozhou	2018	2139	5	3	0	2139	8	183	233.75	140.25	0	374.01	8555.4
Chaozhou	2019	7863	5	6	0	7863	12	326	63.59	76.31	0	152.61	4146
Chaozhou	2020	9398	64	38	2	9398	4	418	681	404.34	21.28	42.56	4447.75
Chaozhou	2021	12064	41	25	1	12064	27	379	339.85	207.23	8.29	223.81	3141.58
Chaozhou	2022	5697	74	29	4	5697	8	343	1298.93	509.04	70.21	140.42	6020.71

Jieyang	2017	6164	36	40	0	6164	15	278	584.04	648.93	0	243.35	4510.06
Jieyang	2018	5934	70	62	1	5934	2	135	1179.64	1044.83	16.85	33.7	2275.03
Jieyang	2019	17190	129	126	3	17190	48	655	750.44	732.98	17.45	279.23	3810.35
Jieyang	2020	25283	258	169	8	25283	27	749	1020.45	668.43	31.64	106.79	2962.46
Jieyang	2021	26348	146	79	3	26348	70	750	554.12	299.83	11.39	265.67	2846.52
Jieyang	2022	8868	58	61	2	8868	9	696	654.04	687.87	22.55	101.49	7848.44
Shantou	2017	533	0	0	0	533	0	0	0	0	0	0	0
Shantou	2018	2185	10	4	0	2185	1	91	457.67	183.07	0	45.77	4164.76
Shantou	2019	6567	22	25	1	6567	1	169	335.01	380.69	15.23	15.23	2573.47
Shantou	2020	14941	86	75	3	14941	19	418	575.6	501.97	20.08	127.17	2797.67
Shantou	2021	12102	70	43	1	12102	39	384	578.42	355.31	8.26	322.26	3173.03
Shantou	2022	5868	62	31	0	5868	11	362	1056.58	528.29	0	187.46	6169.05
Shanwei	2017	2344	7	7	1	2344	24	99	298.63	298.63	42.66	1023.89	4223.55
Shanwei	2018	4709	39	42	0	4709	0	525	828.2	891.91	0	0	11148.86
Shanwei	2019	19067	55	42	3	19067	109	943	288.46	220.28	15.73	571.67	4945.72
Shanwei	2020	12786	59	40	7	12786	82	551	461.44	312.84	54.75	641.33	4309.4
Shanwei	2021	12604	52	33	3	12604	88	567	412.57	261.82	23.8	698.19	4498.57
Shanwei	2022	8175	39	34	0	8175	18	449	477.06	415.9	0	220.18	5492.35
Heyuan	2017	4931	19	32	3	4931	10	286	385.32	648.96	60.84	202.8	5800.04
Heyuan	2018	10220	88	61	6	10220	13	735	861.06	596.87	58.71	127.2	7191.78
Heyuan	2019	19628	145	87	8	19628	13	1234	738.74	443.24	40.76	66.23	6286.94
Heyuan	2020	20457	240	126	13	20457	23	1358	1173.19	615.93	63.55	112.43	6638.31
Heyuan	2021	37736	234	119	13	37736	42	2165	620.1	315.35	34.45	111.3	5737.23
Heyuan	2022	11049	132	59	3	11049	10	1298	1194.68	533.98	27.15	90.51	11747.67
Meizhou	2017	11570	97	57	1	11570	4	258	838.38	492.65	8.64	34.57	2229.9
Meizhou	2018	10100	102	61	1	10100	10	334	1009.9	603.96	9.9	99.01	3306.93

Meizhou	2019	33274	207	223	4	33274	125	1709	622.11	670.19	12.02	375.67	5136.14
Meizhou	2020	27147	177	162	9	27147	0	744	652.01	596.75	33.15	0	2740.63
Meizhou	2021	23152	185	170	6	23152	0	789	799.07	734.28	25.92	0	3407.91
Meizhou	2022	7132	92	58	4	7132	0	321	1289.96	813.24	56.09	0	4500.84
Qingyuan	2017	1986	2	7	0	1986	6	47	100.7	352.47	0	302.11	2366.57
Qingyuan	2018	4702	31	29	0	4702	64	277	659.29	616.76	0	1361.12	5891.11
Qingyuan	2019	17795	164	92	6	17795	227	1334	921.61	517	33.72	1275.64	7496.49
Qingyuan	2020	24056	218	154	5	24056	279	1625	906.22	640.17	20.78	1159.79	6755.07
Qingyuan	2021	16909	253	88	3	16909	443	1303	1496.24	520.43	17.74	2619.91	7705.96
Qingyuan	2022	10236	122	64	2	10236	147	822	1191.87	625.24	19.54	1436.11	8030.48
Shaoguan	2017	5627	26	30	1	5627	61	331	462.06	533.14	17.77	1084.06	5882.35
Shaoguan	2018	7977	94	35	3	7977	51	290	1178.39	438.76	37.61	639.34	3635.45
Shaoguan	2019	15087	153	92	9	15087	77	699	1014.12	609.8	59.65	510.37	4633.13
Shaoguan	2020	27071	422	158	8	27071	59	1248	1558.86	583.65	29.55	217.95	4610.1
Shaoguan	2021	14426	275	88	4	14426	105	747	1906.28	610.01	27.73	727.85	5178.15
Shaoguan	2022	8002	189	67	3	8002	33	604	2361.91	837.29	37.49	412.4	7548.11
Dongguan	2017	69848	316	144	33	NA	NA	NA	452.41	206.16	47.25	NA	NA
Dongguan	2020	190812	819	402	24	NA	NA	NA	429.22	210.68	12.58	NA	NA
Dongguan	2021	66779	338	190	9	NA	NA	NA	506.15	284.52	13.48	NA	NA
Dongguan	2022	49725	186	107	7	NA	NA	NA	374.06	215.18	14.08	NA	NA
Foshan	2018	4673	2	0	0	4673	3	19	42.8	0	0	64.2	406.59
Foshan	2019	20957	15	15	3	20957	70	630	71.58	71.58	14.32	334.02	3006.16
Foshan	2020	24463	3	5	0	24463	198	1527	12.26	20.44	0	809.39	6242.08
Foshan	2021	54573	413	165	3	54573	112	2650	756.78	302.35	5.5	205.23	4855.88
Foshan	2022	2485	36	0	0	2485	0	132	1448.69	0	0	0	5311.87
Guangzhou	2017	78301	464	27	36	NA	NA	NA	592.59	34.48	45.98	NA	NA

Guangzhou	2018	50344	287	5	19	NA	NA	NA	570.08	9.93	37.74	NA	NA
Guangzhou	2019	54449	201	8	10	NA	NA	NA	369.15	14.69	18.37	NA	NA
Guangzhou	2020	60018	245	18	17	NA	NA	NA	408.21	29.99	28.32	NA	NA
Guangzhou	2021	47228	233	14	12	NA	NA	NA	493.35	29.64	25.41	NA	NA
Guangzhou	2022	72244	203	7	4	NA	NA	NA	280.99	9.69	5.54	NA	NA
Huizhou	2017	2399	21	14	1	2399	0	111	875.36	583.58	41.68	0	4626.93
Huizhou	2018	7746	162	53	3	7746	0	375	2091.4	684.22	38.73	0	4841.21
Huizhou	2019	20149	299	117	5	20149	0	652	1483.94	580.67	24.82	0	3235.89
Huizhou	2020	20668	235	148	9	20668	120	1265	1137.02	716.08	43.55	580.61	6120.57
Huizhou	2021	31073	320	85	8	31073	317	1948	1029.83	273.55	25.75	1020.18	6269.11
Huizhou	2022	9087	109	32	2	9087	80	602	1199.52	352.15	22.01	880.38	6624.85
Jiangmen	2018	2089	18	9	0	2089	0	34	861.66	430.83	0	0	1627.57
Jiangmen	2019	10088	81	72	1	10088	1	277	802.93	713.72	9.91	9.91	2745.84
Jiangmen	2020	25412	142	148	9	25412	46	805	558.79	582.4	35.42	181.02	3167.79
Jiangmen	2021	15335	117	84	6	15335	46	329	762.96	547.77	39.13	299.97	2145.42
Jiangmen	2022	2035	35	15	4	2035	2	168	1719.9	737.1	196.56	98.28	8255.53
Zhaoqing	2017	2181	11	4	0	2181	1	51	504.36	183.4	0	45.85	2338.38
Zhaoqing	2018	8144	72	35	3	8144	0	434	884.09	429.76	36.84	0	5329.08
Zhaoqing	2019	25826	227	144	6	25826	3	1496	878.96	557.58	23.23	11.62	5792.61
Zhaoqing	2020	18006	150	116	9	18006	0	1244	833.06	644.23	49.98	0	6908.81
Zhaoqing	2021	17648	169	107	5	17648	0	964	957.62	606.3	28.33	0	5462.38
Zhaoqing	2022	11608	106	65	5	11608	2	856	913.16	559.96	43.07	17.23	7374.22
Zhongshan	2017	29840	37	51	1	NA	NA	NA	123.99	170.91	3.35	NA	NA
Zhongshan	2018	83427	336	178	12	NA	NA	NA	402.75	213.36	14.38	NA	NA
Zhongshan	2019	58024	258	110	7	NA	NA	NA	444.64	189.58	12.06	NA	NA
Zhongshan	2020	36512	338	147	12	NA	NA	NA	925.72	402.61	32.87	NA	NA

Zhongshan	2021	25099	916	53	1	NA	NA	NA	3649.55	211.16	3.98	NA	NA
Zhongshan	2022	25104	789	66	1	NA	NA	NA	3142.93	262.91	3.98	NA	NA
Maoming	2017	7455	51	59	2	7455	2	300	684.1	791.42	26.83	26.83	4024.14
Maoming	2018	17702	208	144	11	17702	4	959	1175.01	813.47	62.14	22.6	5417.47
Maoming	2019	29142	367	226	23	29142	13	1361	1259.35	775.51	78.92	44.61	4670.24
Maoming	2020	32449	450	302	24	32449	12	2571	1386.79	930.69	73.96	36.98	7923.2
Maoming	2021	10159	164	104	6	10159	0	445	1614.33	1023.72	59.06	0	4380.35
Maoming	2022	15203	253	121	11	15203	43	1123	1664.15	795.9	72.35	282.84	7386.7
Yangjiang	2017	2145	14	17	1	2145	0	56	652.68	792.54	46.62	0	2610.72
Yangjiang	2018	3716	33	15	4	3716	0	77	888.05	403.66	107.64	0	2072.12
Yangjiang	2019	27843	142	113	5	27843	0	143	510	405.85	17.96	0	513.59
Yangjiang	2020	10012	99	54	4	10012	0	168	988.81	539.35	39.95	0	1677.99
Yangjiang	2021	11420	103	41	2	11420	2	156	901.93	359.02	17.51	17.51	1366.02
Yangjiang	2022	7199	74	37	1	7199	6	234	1027.92	513.96	13.89	83.34	3250.45
Yunfu	2017	749	2	2	0	749	1	8	267.02	267.02	0	133.51	1068.09
Yunfu	2018	6583	48	24	2	6583	0	244	729.15	364.58	30.38	0	3706.52
Yunfu	2019	20271	255	129	6	20271	6	809	1257.95	636.38	29.6	29.6	3990.92
Yunfu	2020	12197	256	108	1	12197	0	476	2098.88	885.46	8.2	0	3902.6
Yunfu	2021	10520	123	36	1	10520	0	316	1169.2	342.21	9.51	0	3003.8
Yunfu	2022	5512	100	36	0	5512	1	338	1814.22	653.12	0	18.14	6132.08
Zhanjiang	2017	3989	23	9	0	3989	8	260	576.59	225.62	0	200.55	6517.92
Zhanjiang	2018	10368	43	29	0	10368	30	503	414.74	279.71	0	289.35	4851.47
Zhanjiang	2019	44265	171	157	9	44265	104	1870	386.31	354.68	20.33	234.95	4224.56
Zhanjiang	2020	34925	131	162	14	34925	112	2241	375.09	463.85	40.09	320.69	6416.61
Zhanjiang	2021	22024	132	96	8	22024	52	980	599.35	435.89	36.32	236.11	4449.69
Zhanjiang	2022	9654	65	45	7	9654	40	726	673.3	466.13	72.51	414.34	7520.2

Cervical lesion proportions were calculated using the annual number of screening records as the denominator, whereas HPV positivity proportions were calculated using the annual number of HPV-tested records. All proportions were crude and expressed per 100,000 records. Missing observations were not imputed. AIS, adenocarcinoma in situ; HPV, human papillomavirus; HSIL, high-grade squamous intraepithelial lesion; LSIL, low-grade squamous intraepithelial lesion; MIC, microinvasive squamous cell carcinoma or adenocarcinoma; NA, not available.

Table S3. Year-specific HPV positivity, genotype detection, and annual percentage changes in Guangdong Province, 2017-2022.

HPV type	2017	2018	2019	2020	2021	2022	APC (%)	Lower 95%CI	Upper 95%CI
Screening records, n	52410	108987	335012	339271	328093	127810			
Low-risk HPV	132 (0.3)	186 (0.2)	809 (0.2)	981 (0.3)	1343 (0.4)	410 (0.3)	19.21[a]	4.18	44.34
High-risk HPV	2128 (4.1)	5215 (4.8)	14307 (4.3)	17408 (5.1)	14872 (4.5)	9074 (7.1)	8.05[a]	2.53	14.73
Screening records, n (Specific HPV genotype)	50244	104435	319395	326120	319200	125372			
HPV6 (%)	34 (0.1)	50 (0.0)	177 (0.1)	235 (0.1)	307 (0.1)	122 (0.1)	18.5[a]	7.1	34.2
HPV11 (%)	15 (0.0)	20 (0.0)	65 (0.0)	61 (0.0)	82 (0.0)	45 (0.0)	10.1	-10.0	41.0
HPV42 (%)	7 (0.0)	26 (0.0)	129 (0.0)	139 (0.0)	226 (0.1)	80 (0.1)	29.3[a]	12.1	56.1
HPV43 (%)	18 (0.0)	20 (0.0)	104 (0.0)	108 (0.0)	140 (0.0)	52 (0.0)	12.0	-4.3	37.7
HPV44 (%)	3 (0.0)	17 (0.0)	106 (0.0)	114 (0.0)	146 (0.0)	94 (0.1)	35.2[a]	17.8	60.7
HPV81 (%)	55 (0.1)	44 (0.0)	206 (0.1)	282 (0.1)	397 (0.1)	117 (0.1)	14.7	-6.5	49.0
HPV16 (%)	581 (1.2)	1347 (1.3)	2904 (0.9)	2969 (0.9)	2201 (0.7)	1343 (1.1)	-6.9	-23.5	15.0
HPV18 (%)	279 (0.6)	706 (0.7)	1325 (0.4)	1461 (0.4)	1134 (0.4)	760 (0.6)	-3.9	-29.3	34.5
HPV31 (%)	104 (0.2)	271 (0.3)	167 (0.1)	152 (0.0)	163 (0.1)	223 (0.2)	-13.3	-60.9	85.2
HPV33 (%)	118 (0.2)	301 (0.3)	171 (0.1)	211 (0.1)	201 (0.1)	237 (0.2)	-14.2	-60.0	81.8
HPV35 (%)	83 (0.2)	265 (0.3)	171 (0.1)	147 (0.0)	120 (0.0)	202 (0.2)	-14.2	-55.5	60.2
HPV39 (%)	70 (0.1)	129 (0.1)	439 (0.1)	532 (0.2)	458 (0.1)	157 (0.1)	0.8	-7.2	10.7

HPV45 (%)	62 (0.1)	230 (0.2)	73 (0.0)	82 (0.0)	101 (0.0)	174 (0.1)	-13.6	-64.0	101.2
HPV51 (%)	118 (0.2)	130 (0.1)	430 (0.1)	654 (0.2)	584 (0.2)	200 (0.2)	2.5	-20.6	44.4
HPV52 (%)	429 (0.9)	602 (0.6)	1291 (0.4)	1759 (0.5)	1638 (0.5)	867 (0.7)	0.4	-16.8	25.9
HPV53 (%)	92 (0.2)	87 (0.1)	425 (0.1)	566 (0.2)	500 (0.2)	192 (0.2)	4.5	-15.2	36.9
HPV56 (%)	93 (0.2)	122 (0.1)	385 (0.1)	625 (0.2)	350 (0.1)	132 (0.1)	-5.1	-21.8	16.9
HPV58 (%)	214 (0.4)	449 (0.4)	700 (0.2)	783 (0.2)	761 (0.2)	391 (0.3)	-7.9	-29.3	23.9
HPV59 (%)	51 (0.1)	111 (0.1)	342 (0.1)	442 (0.1)	224 (0.1)	190 (0.2)	2.4	-18.0	31.0
HPV66 (%)	37 (0.1)	99 (0.1)	325 (0.1)	379 (0.1)	245 (0.1)	138 (0.1)	0.0	-14.5	19.6
HPV68 (%)	129 (0.3)	212 (0.2)	587 (0.2)	704 (0.2)	529 (0.2)	293 (0.2)	-6.9	-23.5	15.0

Values are n (%) unless otherwise indicated. Overall low-risk and high-risk HPV positivity percentages were calculated using all screening records within each year as denominators, whereas genotype-specific detection used records with genotype-level information. Records with multiple infections contributed once to each detected genotype. Superscript a indicates an APC significantly different from zero at P<0.05. APC, annual percentage change; CI, confidence interval; HPV, human papillomavirus.

Table S4. Region- and age-specific distribution of cervical diagnoses and HPV positivity across screening records in Guangdong Province, 2017-2022.

	Age Group	<45	P_1	45-59	P_2	60-64	P_3	≥65
**Eastern Guangdong, n**		84141		122521		18340		4164
Diagnosis (%)	Normal cervix	82860 (98.48)	0.121	120406 (98.27)	0.63	17958 (97.92)	0.288	4088 (98.17)
	Inflammation	408 (0.48)	0.02	746 (0.61)	0.27	134 (0.73)	0.925	31 (0.74)
	LSIL	532 (0.63)	0.307	721 (0.59)	0.484	115 (0.63)	0.356	21 (0.50)
	HSIL/AIS/MIC	318 (0.38)	0.296	571 (0.47)	0.894	106 (0.58)	0.446	20 (0.48)
	Cervical cancer	8 (0.01)	0.023	26 (0.02)	0.252	7 (0.04)	0.774	2 (0.05)
	Others	15 (0.02)	0.17	51 (0.04)	0.842	20 (0.11)	0.255	2 (0.05)
Low-risk HPV (%)	Negative	83975 (99.80)		122165 (99.71)		18264 (99.59)		4140 (99.42)
	Positive	166 (0.20)	<0.001	356 (0.29)	<0.001	76 (0.41)	0.156	24 (0.58)
High-risk HPV (%)	Negative	81040 (96.31)		117358 (95.79)		17316 (94.42)		3939 (94.60)
	Positive	3101 (3.69)	<0.001	5163 (4.21)	<0.001	1024 (5.58)	0.647	225 (5.40)

**Western Guangdong, n**		107797		204304		34139		9262
Diagnosis (%)	Normal cervix	105482 (97.85)	<0.001	199679 (97.74)	<0.001	33210 (97.28)	0.225	8988 (97.04)
	Inflammation	625 (0.58)	<0.001	1389 (0.68)	<0.001	368 (1.08)	0.690	95 (1.03)
	LSIL	1069 (0.99)	0.351	1867 (0.91)	0.823	289 (0.85)	0.703	82 (0.89)
	HSIL/AIS/MIC	577 (0.54)	<0.001	1188 (0.58)	0.001	221 (0.65)	0.286	80 (0.86)
	Cervical cancer	20 (0.02)	0.001	88 (0.04)	0.071	26 (0.08)	0.681	8 (0.09)
	Others	24 (0.02)	<0.001	93 (0.05)	0.044	25 (0.07)	0.528	9 (0.10)
Low-risk HPV (%)	Negative	107690 (99.90)	0.007	204042 (99.87)	0.055	34091 (99.86)	0.178	9243 (99.79)
	Positive	107 (0.10)		262 (0.13)		48 (0.14)		19 (0.21)
High-risk HPV (%)	Negative	103267 (95.80)	<0.001	195052 (95.47)	<0.001	32132 (94.12)	0.235	8687 (93.79)
	Positive	4530 (4.20)		9252 (4.53)		2007 (5.88)		575 (6.21)
**Northern Guangdong, n**		115011		218239		29447		7573
Diagnosis (%)	Normal cervix	112363 (97.70)	0.005	212930 (97.57)	0.037	28518 (96.85)	0.127	7360 (97.19)
	Inflammation	731 (0.64)	<0.001	1777 (0.81)	0.001	361 (1.23)	0.769	89 (1.18)
	LSIL	1156 (1.01)	0.120	2142 (0.98)	0.171	307 (1.04)	0.091	62 (0.82)
	HSIL/AIS/MIC	679 (0.59)	0.877	1187 (0.54)	0.751	210 (0.71)	0.184	43 (0.57)
	Cervical cancer	15 (0.01)	0.001	76 (0.03)	0.058	18 (0.06)	0.612	6 (0.08)
	Others	67 (0.06)	0.001	127 (0.06)	<0.001	33 (0.11)	0.200	13 (0.17)
Low-risk HPV (%)	Negative	114553 (99.60)	0.002	217139 (99.50)	0.100	29252 (99.34)	0.937	7524 (99.35)
	Positive	458 (0.40)		1100 (0.50)		195 (0.66)		49 (0.65)
High-risk HPV (%)	Negative	108983 (94.76)	<0.001	206329 (94.54)	<0.001	27360 (92.91)	0.900	7040 (92.96)
	Positive	6028 (5.24)		11910 (5.46)		2087 (7.09)		533 (7.04)
**Pearl River Delta, n**		128394		181603		21873		4775

Diagnosis (%)	Normal cervix	125929 (98.08)	0.114	178010 (98.02)	0.2	21351 (97.61)	0.548	4668 (97.76)
	Inflammation	853 (0.66)	0.691	1277 (0.70)	0.942	199 (0.91)	0.184	34 (0.71)
	LSIL	1045 (0.81)	0.65	1469 (0.81)	0.675	193 (0.88)	0.384	36 (0.75)
	HSIL/AIS/MIC	538 (0.42)	0.005	754 (0.42)	0.004	108 (0.49)	0.089	33 (0.69)
	Cervical cancer	19 (0.01)	<0.001	51 (0.03)	0.027	8 (0.04)	0.164	4 (0.08)
	Others	10 (0.01)	0.542	42 (0.02)	0.293	14 (0.06)	0.08	0 (0.00)
Low-risk HPV (%)	Negative	127993 (99.69)		181090 (99.72)		21794 (99.64)		4767 (99.83)
	Positive	401 (0.31)	0.076	513 (0.28)	0.138	79 (0.36)	0.034	8 (0.17)
High-risk HPV (%)	Negative	122017 (95.03)		172941 (95.23)		20587 (94.12)		4531 (94.89)
	Positive	6377 (4.97)	0.655	8662 (4.77)	0.277	1286 (5.88)	0.038	244 (5.11)

Values are n (column %) unless otherwise indicated. Percentages were calculated using the corresponding region–age-group totals as denominators. One participant could contribute more than one screening record. Within each region, P1, P2, and P3 compare the groups aged <45 years, 45-59 years, and 60-64 years, respectively, with the group aged ≥65 years. P values were calculated using the chi-square test or Fisher’s exact test, as appropriate. After Bonferroni correction, a two-sided P value <0.017 was considered statistically significant. AIS, adenocarcinoma in situ; HPV, human papillomavirus; HSIL, high-grade squamous intraepithelial lesion; LSIL, low-grade squamous intraepithelial lesion; MIC, microinvasive squamous cell carcinoma or adenocarcinoma.

Table S5. Comparison of the primary and deduplicated sensitivity analyses for adjusted age-specific associations.

**Outcome**	**Age group (Ref: <45 years)**	**Main analysis RR (95% CI)**	**Sensitivity analysis RR (95% CI)**
**LSIL**	45-59	0.98 (0.94, 1.03)	0.98 (0.93, 1.02)
	60-64	1.04 (0.96, 1.13)	1.03 (0.95, 1.12)
	≥65	1.00 (0.81, 1.24)	1.00 (0.81, 1.24)
**HSIL/AIS/MIC**	45-59	1.07 (1.01, 1.14)	1.08 (1.01, 1.14)
	60-64	1.35 (1.21, 1.49)	1.35 (1.22, 1.50)
	≥65	1.52 (1.22, 1.91)	1.53 (1.23, 1.92)
**Cervical cancer**	45-59	2.09 (1.54, 2.84)	2.11 (1.56, 2.87)

Outcome	Age group (Ref: <45 years)	Main analysis RR (95% CI)	Sensitivity analysis RR (95% CI)
	60-64	3.22 (2.13, 4.86)	3.26 (2.16, 4.92)
	≥65	5.02 (2.43, 10.35)	5.09 (2.47, 10.50)
**Low-risk HPV**	45-59	1.26 (1.16, 1.36)	1.26 (1.16, 1.36)
	60-64	1.65 (1.45, 1.88)	1.65 (1.45, 1.89)
	≥65	2.42 (1.80, 3.26)	2.42 (1.80, 3.26)
**High-risk HPV**	45-59	1.07 (1.04, 1.09)	1.07 (1.04, 1.09)
	60-64	1.39 (1.35, 1.44)	1.39 (1.34, 1.44)
	≥65	1.51 (1.39, 1.63)	1.51 (1.39, 1.63)

Participants aged <45 years were the reference group. Models were adjusted for contraceptive use, ethnicity, menopausal status, parity, reported prior screening history, urban-rural residence, and screening year. The sensitivity analysis retained the earliest eligible record for each identifiable woman. AIS, adenocarcinoma in situ; CI, confidence interval; HPV, human papillomavirus; HSIL, high-grade squamous intraepithelial lesion; LSIL, low-grade squamous intraepithelial lesion; MIC, microinvasive squamous cell carcinoma or adenocarcinoma; RR, relative risk.

Table S6. Region-specific HPV positivity and genotype detection in Guangdong Province, 2017-2022.

HPV type	Eastern Guangdong	Northern Guangdong	Pearl River Delta	Western Guangdong
Screening records, n	229166	370270	336645	355502
Low-risk HPV	622 (0.3)	1802 (0.5)	1001 (0.3)	436 (0.1)
High-risk HPV	9513 (4.2)	20558 (5.6)	16569 (4.9)	16364 (4.6)
Screening records, n	222205	354105	327824	340632
(Specific HPV genotype)				
HPV6 (%)	129 (0.1)	458 (0.1)	148 (0.0)	190 (0.1)
HPV11 (%)	52 (0.0)	104 (0.0)	64 (0.0)	68 (0.0)
HPV42 (%)	84 (0.0)	288 (0.1)	193 (0.1)	42 (0.0)

HPV43 (%)	70 (0.0)	172 (0.0)	169 (0.1)	31 (0.0)
HPV44 (%)	16 (0.0)	306 (0.1)	111 (0.0)	47 (0.0)
HPV81 (%)	256 (0.1)	388 (0.1)	347 (0.1)	110 (0.0)
HPV16 (%)	1568 (0.7)	3903 (1.1)	2612 (0.8)	3262 (1.0)
HPV18 (%)	854 (0.4)	1790 (0.5)	1410 (0.4)	1611 (0.5)
HPV31 (%)	309 (0.1)	267 (0.1)	152 (0.0)	352 (0.1)
HPV33 (%)	329 (0.1)	322 (0.1)	185 (0.1)	403 (0.1)
HPV35 (%)	250 (0.1)	283 (0.1)	123 (0.0)	332 (0.1)
HPV39 (%)	350 (0.2)	657 (0.2)	341 (0.1)	437 (0.1)
HPV45 (%)	220 (0.1)	159 (0.0)	111 (0.0)	232 (0.1)
HPV51 (%)	369 (0.2)	750 (0.2)	511 (0.2)	486 (0.1)
HPV52 (%)	1179 (0.5)	2510 (0.7)	1151 (0.4)	1746 (0.5)
HPV53 (%)	457 (0.2)	714 (0.2)	398 (0.1)	293 (0.1)
HPV56 (%)	294 (0.1)	624 (0.2)	441 (0.1)	348 (0.1)
HPV58 (%)	676 (0.3)	1090 (0.3)	576 (0.2)	956 (0.3)
HPV59 (%)	246 (0.1)	433 (0.1)	349 (0.1)	332 (0.1)
HPV66 (%)	275 (0.1)	370 (0.1)	345 (0.1)	233 (0.1)
HPV68 (%)	495 (0.2)	844 (0.2)	519 (0.2)	596 (0.2)

Values are n (%) unless otherwise indicated. Overall low-risk and high-risk HPV positivity percentages were calculated using all screening records within each region as denominators, whereas genotype-specific detection used records with genotype-level information. Records with multiple infections contributed once to each detected genotype. No formal spatial clustering analysis was performed. HPV, human papillomavirus.

Table S7. Age-specific distribution of cervical diagnoses and HPV positivity after deduplication of screening records, Guangdong Province, 2017-2022.

Age Group	Overall	<45	P_1	45-59	P_2	60-64	P_3	≥65
Screening records, n	1267431	428902		711082		101836		25611

Diagnosis (%)	Normal cervix	1240085 (97.84)	420285 (97.99)	<0.001	695720 (97.84)	<0.001	99135 (97.35)	0.644	24945 (97.40)
	Inflammation	8963 (0.71)	2586 (0.60)	<0.001	5095 (0.72)	<0.001	1034 (1.02)	0.5	248 (0.97)
	LSIL	10888 (0.86)	3762 (0.88)	0.094	6048 (0.85)	0.207	879 (0.86)	0.178	199 (0.78)
	HSIL/AIS/MIC	6570 (0.52)	2091 (0.49)	<0.001	3666 (0.52)	<0.001	638 (0.63)	0.307	175 (0.68)
	Cervical cancer	382 (0.03)	62 (0.01)	<0.001	241 (0.03)	<0.001	59 (0.06)	0.247	20 (0.08)
	Others	543 (0.04)	116 (0.03)	<0.001	312 (0.04)	<0.001	91 (0.09)	0.836	24 (0.09)
Low-risk HPV (%)	Negative	1263617 (99.70)	427777 (99.74)		708885 (99.69)		101443 (99.61)		25512 (99.61)
	Positive	3814 (0.30)	1125 (0.26)	<0.001	2197 (0.31)	0.029	393 (0.39)	0.988	99 (0.39)
High-risk HPV (%)	Negative	1205640 (95.12)	409126 (95.39)		676873 (95.19)		95590 (93.87)		24051 (93.91)
	Positive	61791 (4.88)	19776 (4.61)	<0.001	34209 (4.81)	<0.001	6246 (6.13)	0.801	1560 (6.09)

Values are n (%) unless otherwise indicated. Percentages were calculated using the corresponding age-group totals as denominators. The deduplicated analysis retained the earliest eligible record for each identifiable woman and excluded records without participant identifiers. P1, P2, and P3 compare the groups aged <45 years, 45-59 years, and 60-64 years, respectively, with the group aged ≥65 years. P values were calculated using the chi-square test or Fisher's exact test, as appropriate. After Bonferroni correction, a two-sided P value <0.017 was considered statistically significant. AIS, adenocarcinoma in situ; HPV, human papillomavirus; HSIL, high-grade squamous intraepithelial lesion; LSIL, low-grade squamous intraepithelial lesion; MIC, microinvasive squamous cell carcinoma or adenocarcinoma.

Table S8. Cervical diagnoses and HPV positivity by prior screening history and age group after deduplication, Guangdong Province, 2017-2022.

	Age Group	<45	P_1	45-59	P_2	60-64	P_3	≥65
**No prior screening reported, n**		297920		502922		81986		21606
Diagnosis (%)	Normal cervix	291791 (97.94)	<0.001	491718 (97.77)	<0.001	79817 (97.35)	0.958	21033 (97.35)
	Inflammation	1747 (0.59)	<0.001	3546 (0.71)	<0.001	837 (1.02)	0.522	210 (0.97)
	LSIL	2654 (0.89)	0.115	4278 (0.85)	0.317	698 (0.85)	0.354	170 (0.79)
	HSIL/AIS/MIC	1606 (0.54)	<0.001	2951 (0.59)	0.011	514 (0.63)	0.121	156 (0.72)
	Cervical cancer	45 (0.02)	<0.001	200 (0.04)	0.002	54 (0.07)	0.387	18 (0.08)
	Others	77 (0.03)	<0.001	229 (0.05)	0.005	66 (0.08)	0.734	19 (0.09)
Low-risk HPV (%)	Negative	297158 (99.74)		501385 (99.69)		81668 (99.61)		21519 (99.60)

	Positive	762 (0.26)	<0.001	1537 (0.31)	0.012	318 (0.39)	0.757	87 (0.40)
High-risk HPV (%)	Negative	284186 (95.39)		478518 (95.15)		76976 (93.89)		20280 (93.86)
	Positive	13734 (4.61)	<0.001	24404 (4.85)	<0.001	5010 (6.11)	0.886	1326 (6.14)
**Prior screening reported, n**		130982		208160		19850		4005
Diagnosis (%)	Normal cervix	128494 (98.10)	0.054	204002 (98.00)	0.146	19318 (97.32)	0.196	3912 (97.68)
	Inflammation	839 (0.64)	0.017	1549 (0.74)	0.136	197 (0.99)	0.799	38 (0.95)
	LSIL	1108 (0.85)	0.406	1770 (0.85)	0.388	181 (0.91)	0.246	29 (0.72)
	HSIL/AIS/MIC	485 (0.37)	0.287	715 (0.34)	0.162	124 (0.62)	0.261	19 (0.47)
	Cervical cancer	17 (0.01)	0.052	41 (0.02)	0.183	5 (0.03)	0.404	2 (0.05)
	Others	39 (0.03)	0.001	83 (0.04)	0.009	25 (0.13)	0.986	5 (0.12)
Low-risk HPV (%)	Negative	130619 (99.72)		207500 (99.68)		19775 (99.62)		3993 (99.70)
	Positive	363 (0.28)	0.790	660 (0.32)	0.846	75 (0.38)	0.454	12 (0.30)
High-risk HPV (%)	Negative	124940 (95.39)		198355 (95.29)		18614 (93.77)		3771 (94.16)
	Positive	6042 (4.61)	<0.001	9805 (4.71)	<0.001	1236 (6.23)	0.357	234 (5.84)

Values are n (%) unless otherwise indicated. Percentages were calculated using the corresponding age-group total within each reported prior screening-history stratum. The deduplicated dataset retained the earliest eligible record for each identifiable woman. Prior screening history was self-reported and was distinct from repeated participation in the study program. Within each stratum, P1, P2, and P3 compare the groups aged <45 years, 45-59 years, and 60-64 years, respectively, with the group aged ≥65 years. P values were calculated using the chi-square test or Fisher's exact test, as appropriate; a two-sided P value <0.017 was considered statistically significant after Bonferroni correction. AIS, adenocarcinoma in situ; HPV, human papillomavirus; HSIL, high-grade squamous intraepithelial lesion; LSIL, low-grade squamous intraepithelial lesion; MIC, microinvasive squamous cell carcinoma or adenocarcinoma.

Supplementary Figures

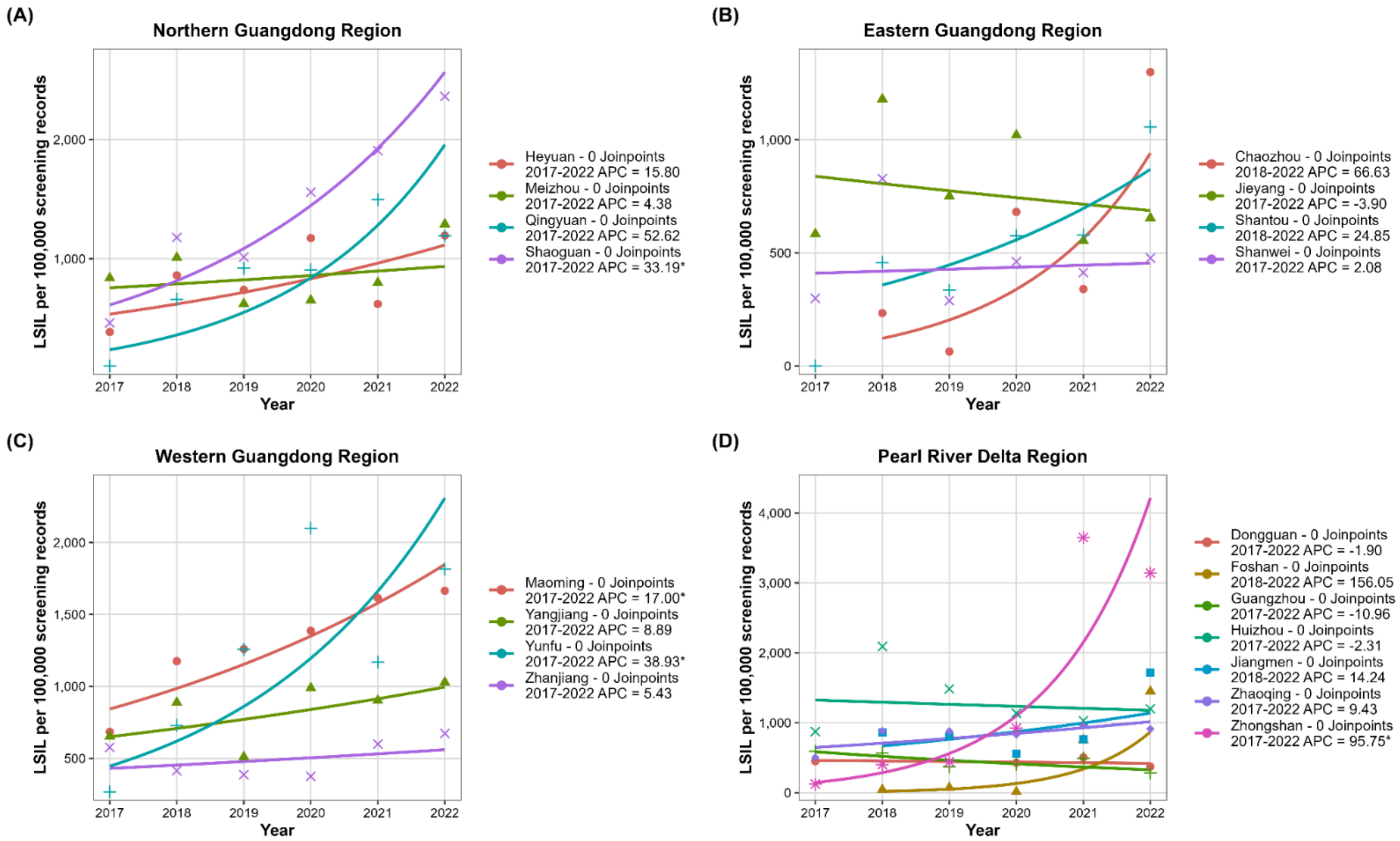

Figure S1. City-specific temporal patterns of crude screening-detected LSIL proportions across regions of Guangdong Province, 2017-2022. (A) Northern Guangdong. (B) Eastern Guangdong. (C) Western Guangdong. (D) Pearl River Delta. Points represent observed annual proportions, and lines represent fitted Joinpoint trends. APC estimates are shown in the panel legends; asterisks indicate P<0.05. Values are expressed per 100,000 screening records. APCs were estimated from available annual data after excluding city-year observations with fewer than 500 screening records. APC, annual percentage change; LSIL, low-grade squamous intraepithelial lesion.

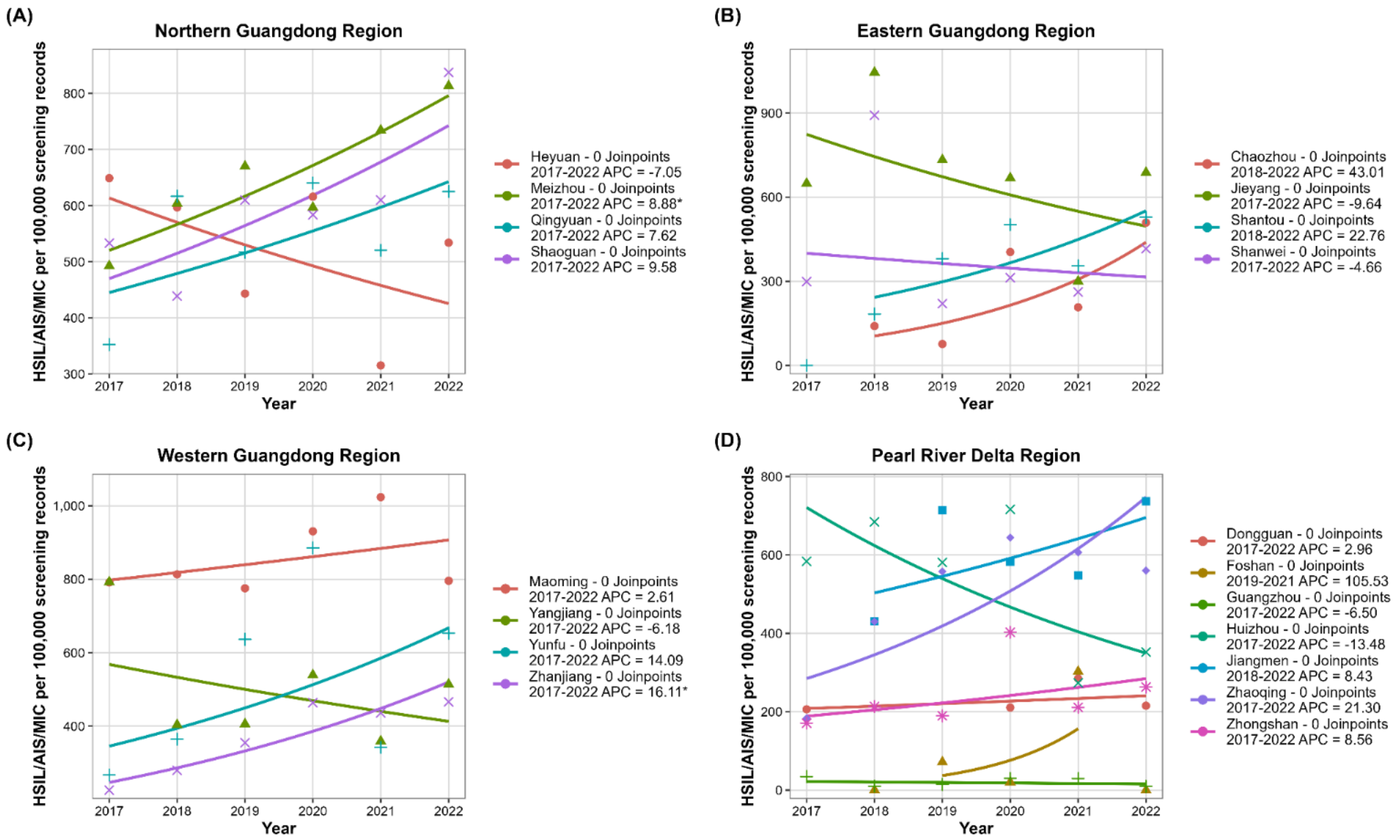

Figure S2. City-specific temporal patterns of crude screening-detected HSIL/AIS/MIC proportions across regions of Guangdong Province, 2017-2022. (A) Northern Guangdong. (B) Eastern Guangdong. (C) Western Guangdong. (D) Pearl River Delta. Points represent observed annual proportions, and lines represent fitted Joinpoint trends. APC estimates are shown in the panel legends; asterisks indicate $P<0.05$. Values are expressed per 100,000 screening records. APCs were estimated from available annual data after excluding city-year observations with fewer than 500 screening records and were not reported when too few reliable observations remained. APC, annual percentage change; AIS, adenocarcinoma in situ; HSIL, high-grade squamous intraepithelial lesion; MIC, microinvasive squamous cell carcinoma or adenocarcinoma.

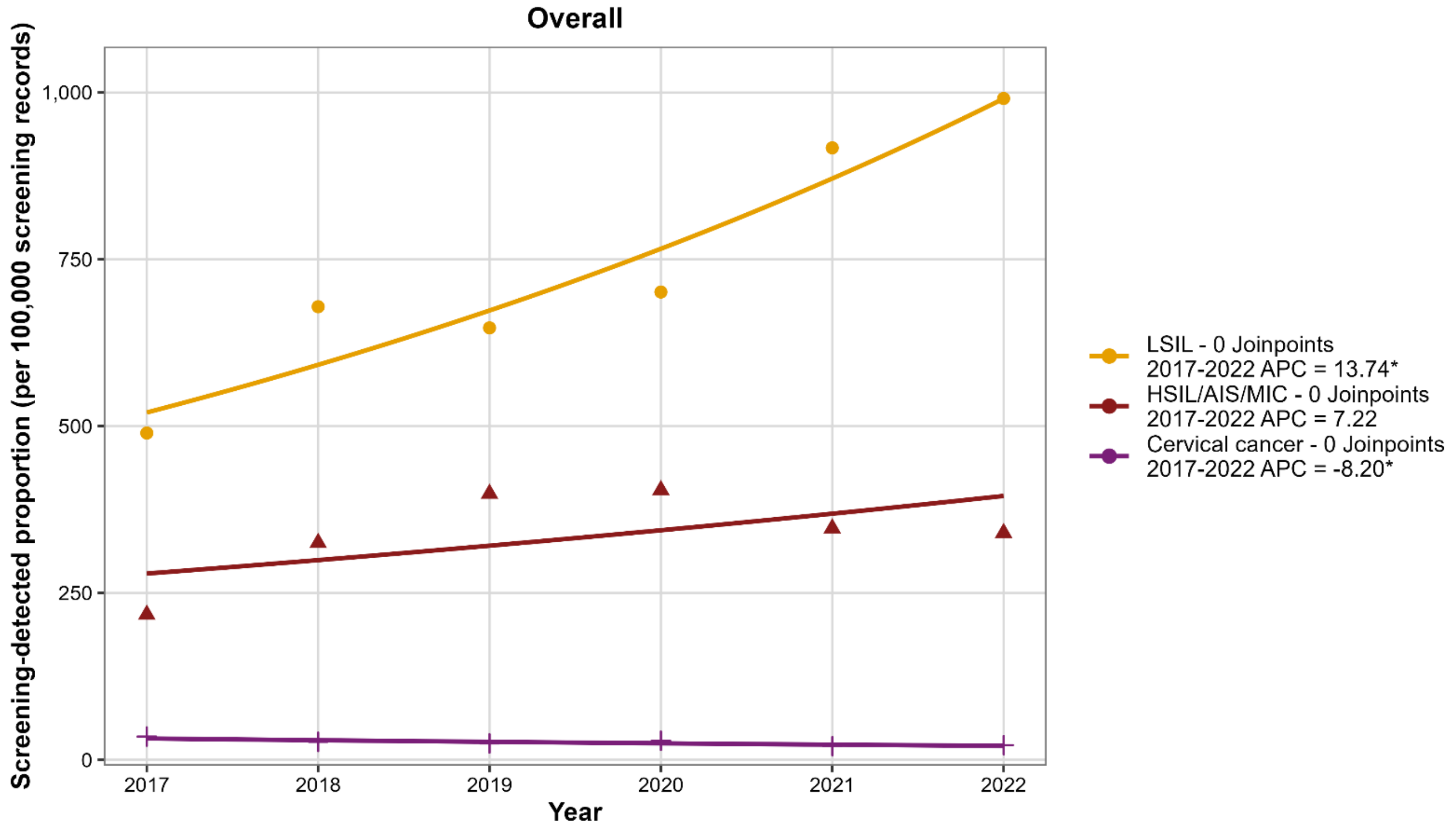

Figure S3. Overall temporal patterns of crude screening-detected cervical lesion proportions in Guangdong Province, 2017-2022. Points represent observed annual proportions, and lines represent fitted Joinpoint trends for LSIL, HSIL/AIS/MIC, and cervical cancer. APC estimates are shown in the figure legend; asterisks indicate $P<0.05$. Values are expressed per 100,000 screening records. APC, annual percentage change; AIS, adenocarcinoma in situ; HSIL, high-grade squamous intraepithelial lesion; LSIL, low-grade squamous intraepithelial lesion; MIC, microinvasive squamous cell carcinoma or adenocarcinoma.
